# Using glycolysis enzyme sequences to inform *Lactobacillus* phylogeny

**DOI:** 10.1099/mgen.0.000187

**Published:** 2018-06-22

**Authors:** Katelyn Brandt, Rodolphe Barrangou

**Affiliations:** ^1^​ Genomic Sciences Graduate Program, North Carolina State University, Raleigh, NC 27695, USA; ^2^​ Department of Food, Bioprocessing and Nutrition Sciences, North Carolina State University, Raleigh, NC 27695, USA

**Keywords:** *Lactobacillus*, phylogeny, glycolysis, evolution

## Abstract

The genus *Lactobacillus* encompasses a diversity of species that occur widely in nature and encode a plethora of metabolic pathways reflecting their adaptation to various ecological niches, including humans, animals, plants and food products. Accordingly, their functional attributes have been exploited industrially and several strains are commonly formulated as probiotics or starter cultures in the food industry. Although divergent evolutionary processes have yielded the acquisition and evolution of specialized functionalities, all *Lactobacillus* species share a small set of core metabolic properties, including the glycolysis pathway. Thus, the sequences of glycolytic enzymes afford a means to establish phylogenetic groups with the potential to discern species that are too closely related from a 16S rRNA standpoint. Here, we identified and extracted glycolysis enzyme sequences from 52 species, and carried out individual and concatenated phylogenetic analyses. We show that a glycolysis-based phylogenetic tree can robustly segregate lactobacilli into distinct clusters and discern very closely related species. We also compare and contrast evolutionary patterns with genome-wide features and transcriptomic patterns, reflecting genomic drift trends. Overall, results suggest that glycolytic enzymes provide valuable phylogenetic insights and may constitute practical targets for evolutionary studies.

## Data Summary

RNA sequencing data has been deposited at the National Center for Biotechnology Information, BioProject PRJNA420353.

**Impact Statement**

Though 16S rRNA-based phylogeny methods have been broadly used, they have a limited ability to precisely ascribe genus species across the prokaryotic branch of the tree of life. In this study, we have shown that using glycolysis enzyme sequences for phylogenetic analyses can be applied to the diverse genus *Lactobacillus*, and is able to consistently unravel phylogenetic groups and precisely ascertain relatedness, even between species nearly identical on the classical ribosomal tree. Because of their universal presence and greater diversity compared to 16S rRNA sequences, we posit that these sequences could be valuable markers in future phylogenetic and microbiome studies, specifically by providing connections to the other major branches, and enabling increased resolution. This can also be used to help identify unknown and un-culturable species, as the glycolysis enzymes are widespread, variable and allow for greater discriminatory power. Importantly, variability within some of the hypervariable regions within glycolytic sequences can also provide discrimination within a species. Looking forward, expanding this analysis to other genera and phylogenetic branches could open new avenues for evolutionary studies, and for investigating the phylogeny, composition and diversity of microbial populations in complex microbiomes.

## Introduction

Genome adaptation is an important feature for speciation, and evolutionary processes balance various adaptive techniques for optimal growth and survival. At the genome level, adaptation features may include gene synteny conservation, G+C mol% drift, as well as codon bias optimization [1–3]. A working balance of these and other forces enable an organism to become uniquely adapted to its niche, and build up competitive advantages in shifting environmental conditions, or overcome predators and competitors. Such unique adaptations are the basis of phylogenetic studies and allow researchers various degrees of discrimination. At the genus and species levels, additions and deletions of genes can be used to define the pan- and core-genome and genome architecture can be used to evaluate synteny [4]. At the strain level, nucleotide polymorphisms afford the highest resolution opportunities, with the ability to compare and contrast nearly identical isolates and even clonal relatives [5, 6].

For prokaryotic species, various tools and methodologies have been used to compare and contrast genomes, but the challenges are often genus- or species-specific, and approaches can vary depending on the desired resolution and encompassed genetic diversity [7]. In some cases where within genus diversity is extensive, such as in bifidobacteria and lactobacilli, using canonical housekeeping genes or universal markers (i.e. 16S rRNA) has proven difficult or limited [8–11]. Also, there has yet to be defined a consistent set of genes to be utilized for multilocus sequence typing studies. Indeed, while universally conserved 16S rRNA sequences afford opportunities for metagenomic analyses, their shortcomings and biases are increasingly under scrutiny [12–14].

For some genera, it has become obvious that the 16S rRNA resolution limit has been met and a new set of criteria must be established. One such genus is *Lactobacillus.* Belonging to the lactic acid bacteria (LAB) group, this genus is composed of over 150 Gram-positive, low G+C species [15, 16]. Lactobacilli have been used as starter cultures in the food industry for decades, and by humankind for millennia, and as such have been labelled generally regarded as safe (GRAS) and benefit from the qualified presumption of safety (QPS) [17]. Food-related studies have led to the assertion that some strains in select species are to be considered probiotic (‘live microorganisms which when administered in adequate amounts confer a health benefit on the host’) [18] and, as such, are now predominantly featured in dairy foods and widely formulated in probiotic dietary supplements [19]. Recently, the advent of microbiome studies has revealed that microbial populations are more numerous, diverse and variable than originally thought [20, 21]. With both qualitative and quantitative considerations, associations and sometimes even correlations have been established between members of the microbiome and host health, though the accuracy and precision with which bacteria are identified vary widely and are not universally satisfactory. One such instance concerns the genus *Lactobacillus*, which has been established as an important colonizer of the human gastrointestinal tract [22]. Additional research is thus needed in this area, as researchers better grasp the role of this genus in health and disease [23–28]. Some lactobacilli are already being exploited, for example, as a tool to deliver vaccines [29]. Arguably, we are far from exhausting all the possible uses of this functional genus. However, in order to be able to fully utilize the numerous functions of *Lactobacillus*, we must first establish a method that enables us to properly identify and relate the many diverse species within this genus. While 16S rRNA sequencing has gotten us this far, it has a limited ability to distinguish between closely related species and represent overall genomic content and reflect genome-wide trends. These shortcomings are certainly not unique to *Lactobacillus,* and with the ever-increasing expansion of our understanding of the microbial world [30], there is a need to identify 16S rRNA-independent genomic features that capture diversity on a more granular level. Thus, it is imperative that a standard method be developed that allows the proper identification of species. In order to achieve this, we assessed the potential of the widespread glycolysis pathway enzyme sequences to inform phylogeny.

In this paper, we applied a previously described method of phylogenetic analysis using the classical glycolysis enzymes as phylogenetic markers [31] to a diverse set of *Lactobacillus* species in order to establish its effect on a complicated genus. Though previous studies had used glycolysis as an expansion of ribosomal trees [32], we determined how a broad glycolysis-based phylogeny compares to the ribosomal tree. Specifically, previous studies have applied glycolysis-based approaches to LAB in order to define an evolutionary pathway. By adding data from the entirety of the glycolysis and pentose phosphate pathways, Salvetti *et al.* [32] were able to apply phenotypic data to explain the branching of the LAB tree, as well as highlight some areas of misclassification in the 16S rRNA tree [32]. Here, we propose using the entirety of the canonical glycolysis pathway as a replacement phylogenetic marker for the 16S rRNA. Conveniently, the glycolysis pathway, much like the 16S rRNA, is universally present, at least partially, conserved, and constitutes a set of suitable candidates for phylogenetic analyses [33, 34]. Here, we demonstrate that this method can assign phylogenetic relationships consistent with what is known from the 16S rRNA marker, though at a much higher discriminatory power. Specifically, we compared sequence-based alignment trees of a representative set of lactobacilli using 16S rRNA- and glycolysis-based approaches. We also analysed the occurrence and location, expression, and G+C mol% of each glycolysis gene. The location and transcriptional profiles confirm that these genes are conserved and highly transcribed with varying levels of drift.

## Methods

### Genomes

We selected 52 diverse species and subspecies of *Lactobacillus* for analysis, sampled across and throughout the 16S rRNA and core- and pan-genome tree (Table 1). We ensured this set was representative of this paraphyletic genus and included species from various niches, as previously established [16]. The genomes were mined using Geneious version 9.0.5 [35] to identify the classical glycolysis genes in each species (Figs S1 and S2, available with the online version of this article). Four reference genomes were used to make a curated database for the glycolysis genes, namely *Lactobacillus acidophilus, Lactobacillus gasseri, Lactobacillus reuteri* and *Lactobacillus rhamnosus.* The Annotate from Database feature was used to annotate the other genomes. To validate the glycolysis annotations, especially in the case of multiple hits, a combination of blast, get_homologues and mRNA-Seq (mRNA sequencing) data was used [36, 37]. The 16S rRNA sequences were extracted from the genomes and blast was used to validate any cases where there were multiple hits. Once annotated and curated, the genes were extracted from the genome. The glycolysis genes were then translated and confirmed by ExPASy [38]. For the concatenated tree, the amino acid sequences were joined together in order of their presence in the glycolysis pathway (Fig. S1).

**Table 1.** Species and genomes list This shows the representative set of 52 *Lactobacillus* species and sub-species used in this study. Accession numbers and naming conventions are included.

**Genus**	**Species**	**Subspecies**	**Strain**	**Accession no.**	**Naming convention**	**Locus tag**
*Lactobacillus*	*acidipiscis*		KCTC 13900	NZ_BACS00000000	L_acidipiscis	GSS
*Lactobacillus*	*acidophilus*		NCFM	NC_006814	L_acidophilus	LBA
*Lactobacillus*	*algidus*		DSM 15638	NZ_AZDI00000000	L_algidus	FC66
*Lactobacillus*	*amylolyticus*		DSM 11664	NZ_ADNY00000000	L_amylolyticus	HMPREF0493
*Lactobacillus*	*amylovorus*		GRL1118	NC_017470	L_amylovorus	LAB52
*Lactobacillus*	*animalis*		DSM 20602	NZ_AEOF00000000	L_animalis	LACAN
*Lactobacillus*	*aquaticus*		DSM 21051	NZ_AYZD00000000	L_aquaticus	FC19
*Lactobacillus*	*brevis*		ATCC 367	NC_008497	L_brevis	LVIS
*Lactobacillus*	*buchneri*		CD034	NC_018610	L_buchneri	LBUCD034
*Lactobacillus*	*cacaonum*		DSM 21116	NZ_AYZE00000000	L_cacaonum	FC80
*Lactobacillus*	*casei*		DSM 20011	NZ_AZCO00000000	L_casei	FC13
*Lactobacillus*	*coryniformis*	*torquens*	DSM 20004	NZ_AEOS00000000	L_coryniformis_t	EWE
*Lactobacillus*	*crispatus*		ST1	NC_014106	L_crispatus	LCRIS
*Lactobacillus*	*curvatus*		CRL 705	NZ_AGBU00000000	L_curvatus	CRL705
*Lactobacillus*	*delbrueckii*	*bulgaricus*	ATCC BAA-365	NC_008529	L_delbrueckii_b	LBUL
*Lactobacillus*	*farciminis*		DSM 20184	NZ_AEOT00000000	L_farciminis	LACFC
*Lactobacillus*	*fermentum*		CECT 5716	NC_017465	L_fermentum	LC40
*Lactobacillus*	*floricola*		DSM 23037	NZ_AYZL00000000	L_floricola	FC86
*Lactobacillus*	*gallinarum*		DSM 10532	NZ_BALB00000000	L_gallinarum	JCM2011
*Lactobacillus*	*gasseri*		ATCC 33323	NC_008530	L_gasseri	LGAS
*Lactobacillus*	*helveticus*		CNRZ32	NC_021744	L_helveticus	LHE
*Lactobacillus*	*hilgardii*		DSM 20176	NZ_ACGP00000000	L_hilgardii	HMPREF0519
*Lactobacillus*	*hominis*		DSM 23910	NZ_CAKE00000000	L_hominis	BN55
*Lactobacillus*	*iners*		DSM 13335	NZ_ACLN00000000	L_iners	HMPREF0520
*Lactobacillus*	*jensenii*		DSM 20557	NZ_AYYU00000000	L_jensenii	FC45
*Lactobacillus*	*johnsonii*		NCC 533	NC_005362	L_johnsonii	LJ
*Lactobacillus*	*kimchicus*		JCM_15530	NZ_AZCX00000000	L_kimchicus	FC96
*Lactobacillus*	*lindneri*		DSM 20690	NZ_JQBT00000000	L_lindneri	IV52
*Lactobacillus*	*mali*		DSM 20444	NZ_AKKT00000000	L_mali	LMA
*Lactobacillus*	*mindensis*		DSM 14500	NZ_AZEZ00000000	L_mindensis	FD29
*Lactobacillus*	*mucosae*		LM1	NZ_CP011013	L_mucosae	LBLM1
*Lactobacillus*	*nasuensis*		JCM_17158	NZ_AZDJ00000000	L_nasuensis	FD02
*Lactobacillus*	*oeni*		DSM 19972	NZ_AZEH00000000	L_oeni	FD46
*Lactobacillus*	*oris*		F0423	NZ_AFTL00000000	L_oris	HMPREF9102
*Lactobacillus*	*otakiensis*		DSM 19908	NZ_BASH00000000	L_otakiensis	LOT
*Lactobacillus*	*parabuchneri*		DSM 5707	NZ_AZGK00000000	L_parabuchneri	FC51
*Lactobacillus*	*paracasei*		N1115	NZ_CP007122	L_paracasei	AF91
*Lactobacillus*	*pasteurii*		DSM 23907	NZ_CAKD00000000	L_pasteurii	BN53
*Lactobacillus*	*pentosus*		DSM 20314	NZ_AZCU00000000	L_pentosus	FD24
*Lactobacillus*	*plantarum*		16	NC_021514	L_plantarum	LP16
*Lactobacillus*	*reuteri*		DSM 20016	NC_009513	L_reuteri	LREU
*Lactobacillus*	*rhamnosus*		GG	NC_013198	L_rhamnosus	LGG
*Lactobacillus*	*rossiae*		DSM 15814	NZ_AZFF00000000	L_rossiae	FD35
*Lactobacillus*	*ruminis*		ATCC 27782	NC_015975	L_ruminis	LRC
*Lactobacillus*	*sakei*	*sakei*	DSM 20017	NZ_BALW00000000	L_sakei_s	JCM1157
*Lactobacillus*	*salivarius*		CECT 5713	NC_017481	L_salivarius	CECT 5713
*Lactobacillus*	*sanfranciscensis*		TMW 1.1304	NC_015978	L_sanfranciscensis	LSA
*Lactobacillus*	*suebicus*		DSM 5007	NZ_BACO00000000	L_suebicus	GSK
*Lactobacillus*	*sunkii*		DSM 19904	NZ_AZEA00000000	L_sunkii	FD17
*Lactobacillus*	*vaginalis*		DSM 5837	NZ_ACGV00000000	L_vaginalis	HMPREF0549
*Lactobacillus*	*versmoldensis*		DSM 14857	NZ_BACR00000000	L_versmoldensis	GSQ
*Lactobacillus*	*zymae*		DSM 19395	NZ_AZDW00000000	L_zymae	FD38

### Transcriptional profiles of glycolysis genes

We analysed RNA transcription profiles from mRNA-Seq data for six species (*L. acidophilus, Lactobacillus amylovorus, Lactobacillus crispatus, Lactobacillus delbrueckii* subsp. *bulgaricus, L. gasseri, and Lactobacillus helveticus*) with the previously published isolation method, mRNA sequencing and analyses [39]. Briefly, we used mRNA-Seq data generated in our laboratory to determine the boundaries and quantitative amounts of RNA transcripts for glycolysis genes as previously described. Samples were grown to mid-log phase and flash-frozen. Single-read RNA sequencing was performed on the extracted RNA using an Illumina HiSeq 2500. Data was then quality assessed, trimmed, filtered and mapped on the reference genomes. Presumably, levels of constitutive transcription reflect biological relevance in the tested conditions and transcript boundaries inform on co-transcribed functional pairs.

### Alignments and trees

Alignments and trees were generated using a previously described methodology [31]. Briefly, once curated sequences were extracted, we aligned the sequences using clustalw (IUB, gap penalty of 15, gap extension of 6.66), muscle (eight iterations), Geneious [global alignment with free end gaps, cost matrix was BLOSUM62 (amino acids) or 65 % similarity (nucleotide)] and mafft [algorithm was auto, scoring matrix was BLOSUM62 and BLOSUM80 (amino acids) or 100PAM and 200PAM (nucleotide), gap penalty of 1.53, offset 0.123], then used trimAl (compareset and automated1) to find a consistent alignment [35, 40–43]. Trees were then generated using RaxML [CAT BLOSUM62 (amino acids) or CAT GTR (nucleotide), Bootstrap using rapid hill climbing with random seed 1, replicates were 100] [44]. A consensus tree was then established using a 50 % threshold level.

### R analyses

Statistical analyses were performed using R version 3.2.2. [45]. R was used to create plots, graphs and quantitative data. Statistical tests used included a two-tailed *t*-test for comparing G+C contents. Default settings were used to preform statistical analyses and assess quantitative distributions.

## Results

### 16S rRNA phylogeny

**Fig. 1.**
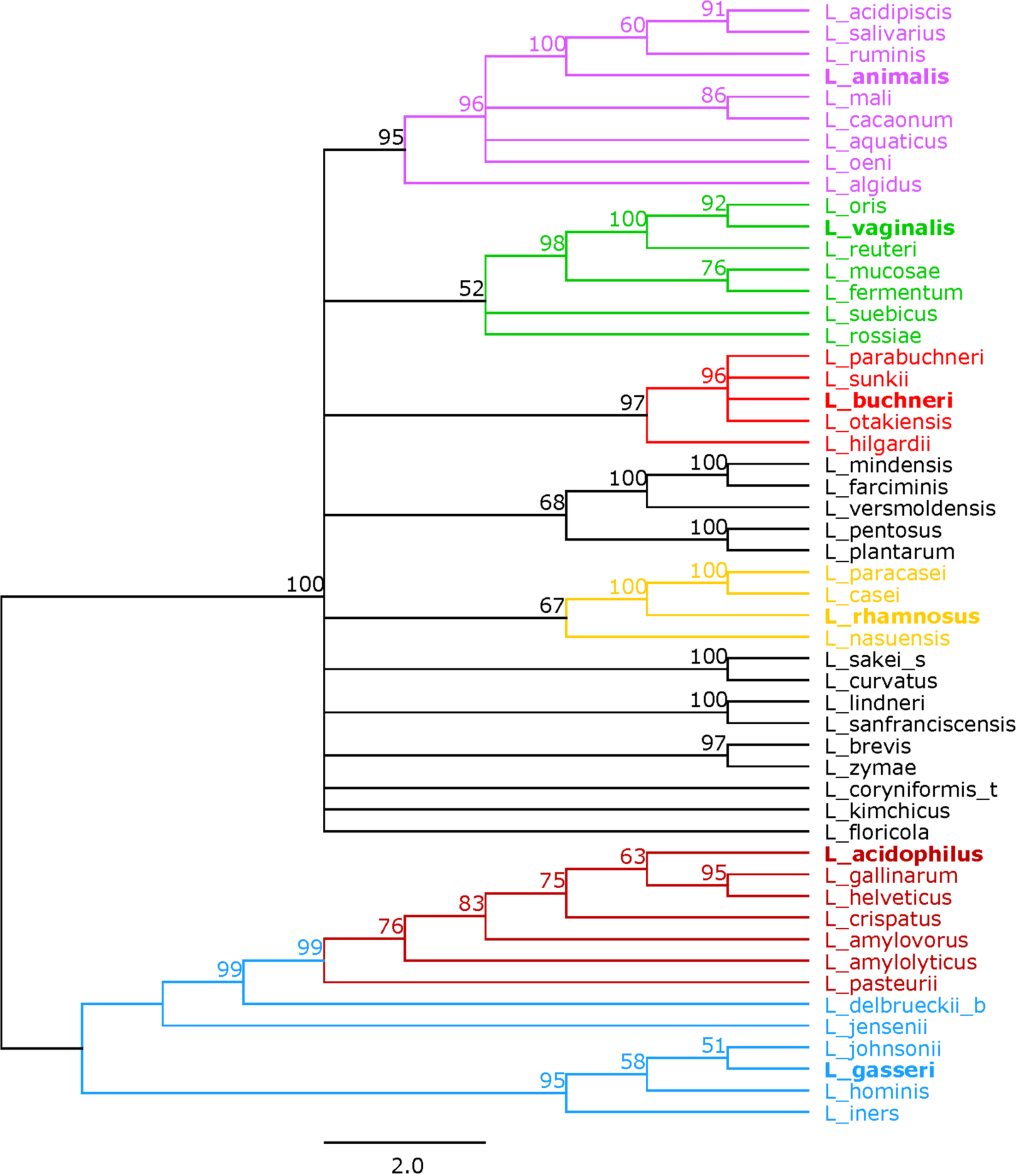
16S rRNA tree. Tree based on the alignment of the 16S rRNA sequences using RaxML. Bootstrap values are recorded on the nodes. Groups are coloured as follows: the *L. animalis* group in purple, the *L. vaginalis* group in green, the *L. buchneri* group in red, the *L. rhamnosus* group in yellow, the *L. acidophilus* group in maroon, and the *L. gasseri* group in blue. The representative species in each group is in bold. Species names follow the naming convention shown in Table 1.

We first generated a 16S rRNA-based tree to use as a reference for our subsequent analyses. A phylogenetic tree based on the alignment of the 16S rRNA sequences from a representative set of 52 species and sub-species of *Lactobacillus* is depicted in Fig. 1. Six phylogenetic groups were identified based on their branching: the *Lactobacillus animalis* group, the *Lactobacillus vaginalis* group, the *Lactobacillus buchneri* group, the *L. rhamnosus* group, the *L. acidophilus* group and the *L. gasseri* group. These groupings are consistent with historically established relationships, as well as recent core-genome analyses [16, 46]. Some of these groups also encompass species that have been historically associated with distinct niches and points of isolation (i.e. mucosal vs intestinal vs dairy origins) [16]. The groups ranged in size from four to nine genomes with the *L. rhamnosus* group as the smallest and the *L. animalis* group as the largest. The bootstrap values for the 16S rRNA tree ranged from 51 to 100. There were 27 nodes that had a bootstrap of 70 or greater (Fig. S3). We used these six phylogenetic groups as references for our subsequent analyses, though some species were not assigned to one of these six groups.

### Glycolysis gene expression

Before using the glycolysis enzymes as phylogenetic markers, we first explored their genetic properties in *Lactobacillus*. Of the 52 *Lactobacillus* species and sub-species selected, 35 species encoded all ten of the classical glycolytic genes. In contrast, 16 species (encompassing the *L. vaginalis* and *L. buchneri* groups) presented eight of the canonical genes (missing *pfk* and *fba*) (Fig. S2). In such cases, alternative metabolic pathways may be utilized, such as the pentose phosphate pathway (*Lactobacillus fermentum*) or the phosphoketolase pathway (*L. buchneri*) [47, 48]. *L. reuteri* uses a mixture of the Embden–Meyerhof pathway and phosphoketolase pathway and, thus, was the only species with six of the glycolysis genes (Fig. S2) [49].

**Fig. 2.**
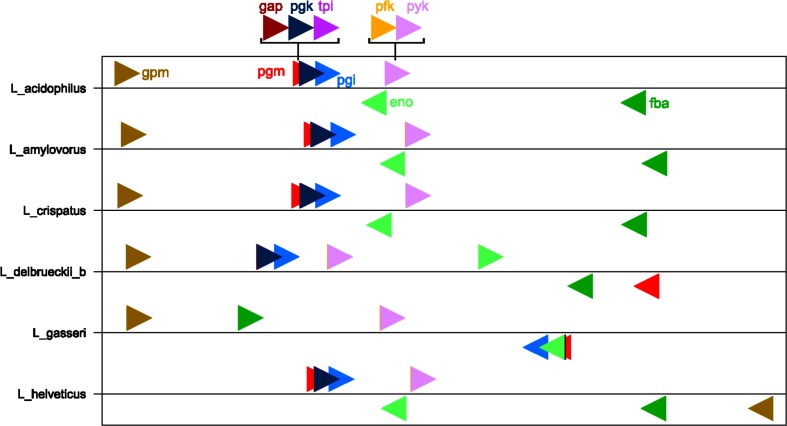
Genomic location. Normalized glycolysis gene locations in *L. acidophilus, L. amylovorus, L. crispatus, L. delbrueckii* subsp. *bulgaricus*, *L. gasseri* and *L. helveticus.* Normalization was calculated by dividing the location on the genome by the total genome size. Right arrows indicate forward direction, left reverse direction. The genomes are organized in the 5′ to 3′ direction. Colours are as follows: *pgm* in red, *pgi* in blue, *pfk* in yellow, *fba* in dark green, *tpi* in purple, *gap* in maroon, *pgk* in navy, *gpm* in mustard, *eno* in light green and *pyk* in lavender.

**Fig. 3.**
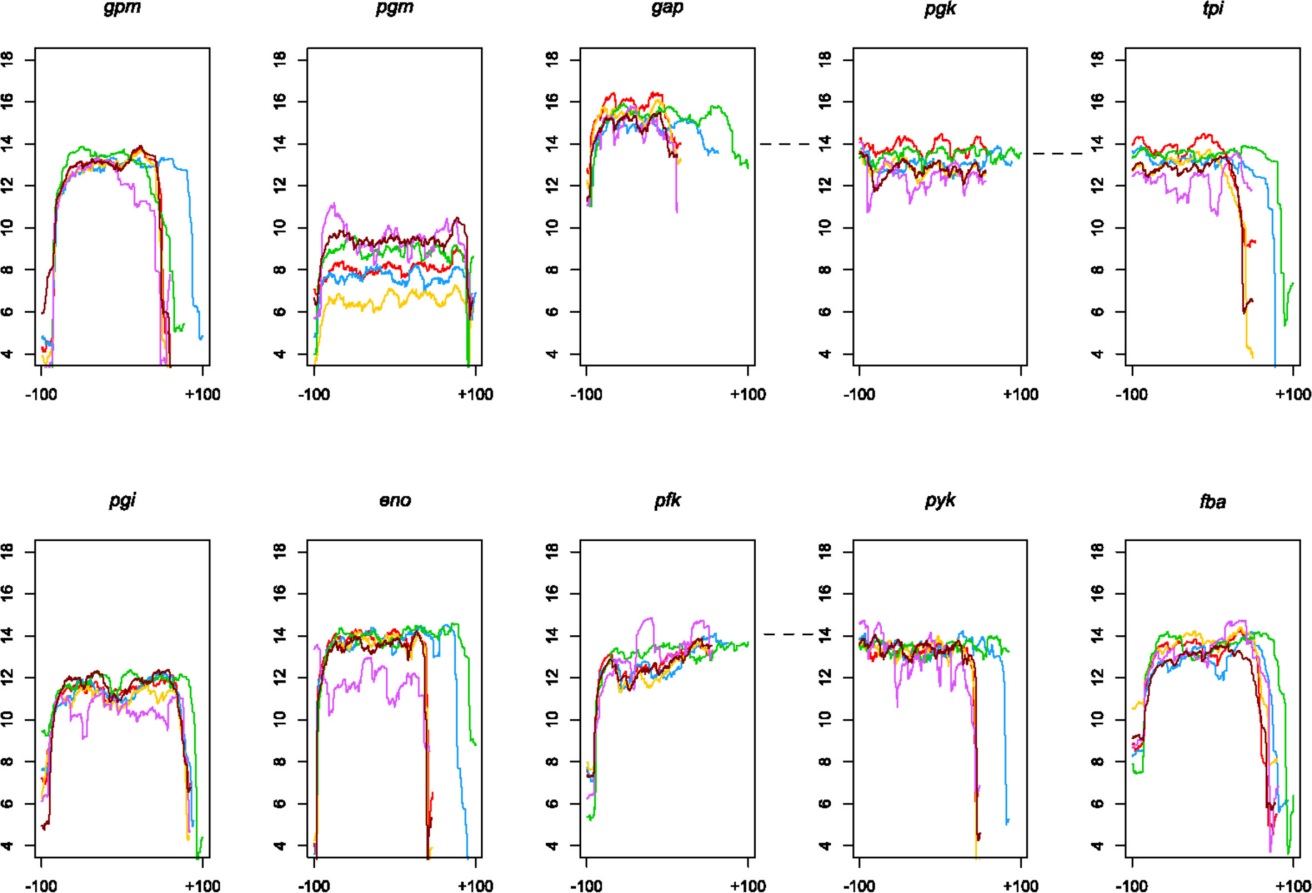
Glycolysis genes transcription. Each plot represents the mRNA-Seq coverage, log2 transformed, for the corresponding glycolysis gene over its length; ±100 represents the number of bases away from the start/end of the gene. The species are plotted as follows: *L. acidophilus* is red, *L. amylovorus* in blue, *L. crispatus* in yellow, *L. delbrueckii* subsp. *bulgaricus* in green, *L. gasseri* in purple and *L. helveticus* in maroon.

**Fig. 4.**
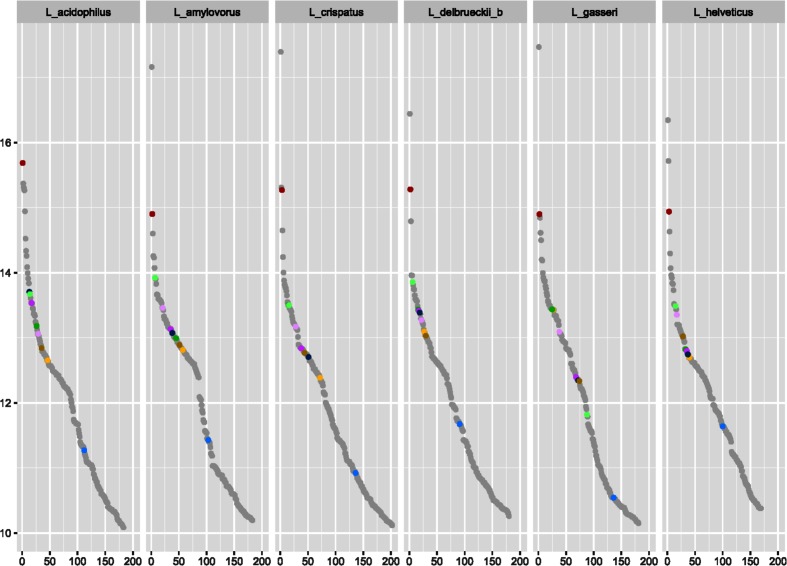
Ranked order of mRNA expression. Top 10 % most highly expressed genes in *L. acidophilus, L. amylovorus, L. crispatus, L. delbrueckii* subsp. *bulgaricus*, *L. gasseri* and *L. helveticus.* Data is represented as a log2 transformed RPKM (Reads Per Kilobase of transcript, per Million mapped reads). Transcripts are ranked from most abundant to least abundant. Glycolysis genes are coloured as follows: *pgm* in red, *pgi* in blue, *pfk* in yellow, *fba* in dark green, *tpi* in purple, *gap* in maroon, *pgk* in navy, *gpm* in mustard, *eno* in light green and *pyk* in lavender.

Next, we characterized the transcripts of glycolysis genes in *Lactobacillus*. Chromosome location and mRNA sequence data were analysed from six species: *L. acidophilus*, *L. amylovorus*, *L. crispatus*, *L. delbrueckii* subsp. *bulgaricus*, *L. gasseri* and *L. helveticus*. These six species fall into the *L. acidophilus* and *L. gasseri* groups, and all six species contain the complete complement of glycolysis genes, allowing for inferences on all of the genes in this study, instead of just a subset. Fig. 2 depicts the location of the glycolysis genes on normalized chromosomes for each of these six species. It is noteworthy that two operons can be visualized: the *gap*, *pgk* and *tpi* operon, as well as the *pfk* and *pyk* operon. Furthermore, the operon boundaries are clearly seen in the mRNA coverage data for each of the six species (Fig. 3). The remaining five genes have clear start and stop boundaries. Notably, *L. helveticus* has a unique arrangement of the glycolysis genes compared to the other five species, possibly due to the large number of IS elements leading to genome decay; however, the operons remain conserved [50]. Next, we compared the expression levels of the glycolysis genes to the whole transcriptome. We found that the glycolysis genes are among the most highly expressed genes. Indeed, considering the top 10 % of the most highly expressed genes in the cell, nine of the ten glycolysis genes are listed (Fig. 4). The only gene absent from the top 10 % is *pgm*. Strikingly, the *gap* gene is consistently among the top three most highly expressed genes in all six species. Such a consistently high transcription level indicates that the *gap* gene is critical to the functionality of the cell and perhaps, as such, less susceptible to changes. This is also reflected by the conserved location of *gap* in the genome and operon structure amongst the strains studied (Fig. 2), potentially indicating uses for *gap* in identification. These results demonstrate that glycolysis genes are genomically conserved, organizationally syntenous and transcriptionally important, showcasing their use as potential phylogenetic markers.

### Glycolysis-based phylogeny

**Fig. 5.**
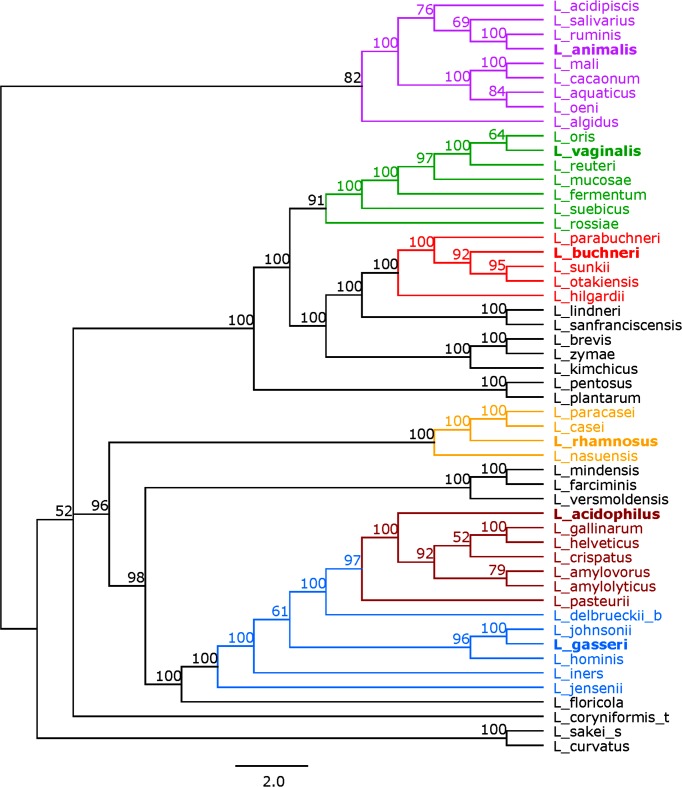
Concatenated glycolysis tree. Tree based on the alignment of concatenated amino acid sequences of glycolysis enzymes using RaxML. Bootstrap values are recorded on the nodes. Groups are coloured as follows: the *L. animalis* group in purple, the *L. vaginalis* group in green, *L. buchneri* group in red, the *L. rhamnosus* group in yellow, the *L. acidophilus* group in maroon, and the *L. gasseri* group in blue. The representative species in each group is in bold. Species name follows the naming convention shown in Table 1.

To create a glycolysis-based phylogeny for the 52 selected *Lactobacillus* species and subspecies, the concatenated amino acid sequences of the glycolysis enzymes were used (Fig. 5). The enzymes were concatenated in their order of occurrence in the glycolysis pathway (Fig. S1). For organisms with all enzymes present, this meant ten sequences were concatenated together, whereas only six to eight amino acid sequences were concatenated for the other species (Fig. S2). The six phylogenetic groups identified from the 16S rRNA reference tree, namely *L. animalis, L. vaginalis, L. buchneri, L. rhamnosus, L. acidophilus* and *L. gasseri*, were also identified in the concatenated tree and follow the same clustering (colouring) scheme. The bootstrap values for the concatenated tree ranged from 52 to 100. Nodes with bootstrap values equal to or greater than 70 numbered 43, a 59 % increase from that of the 16S rRNA tree. Overall, the concatenated tree correctly assigned the phylogenetic groups established from the 16S rRNA tree. In addition, the concatenated tree better discerned how the phylogenetic groups relate to one another, even within groups. This is supported by the higher bootstrap values (Fig. S3). Trees based on the individual glycolysis enzymes can be found in Figs S4–S13. The sum of branch lengths for each tree can be found in Table S1. A detailed comparative analysis of various trees structures revealed that overall there is high congruence in clustering both between and within the six established groups, though with various levels of discrimination across each protein sequence. Repeatedly, glycolysis-based trees provided more discriminatory power than the 16S rRNA tree.

### G+C content analyses

**Fig. 6.**
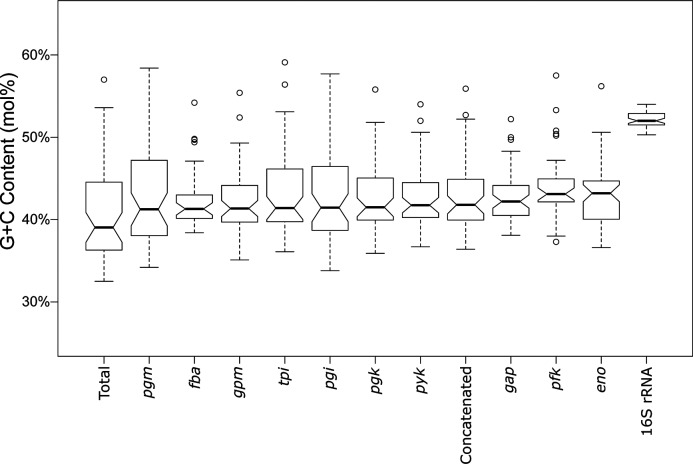
G+C mol% analysis of *Lactobacillus* glycolysis genes. Depicted are notched boxplots of G+C mol% for each glycolysis gene, concatenated genes, 16S rRNA and total genome. Genes are placed in order of increasing median. If two notches do not overlap, it is an indication of strong evidence for differing medians.

Next, we looked at the G+C mol% and genomic drift of the glycolysis genes across the various species. Fig. 6 shows notched boxplots comparing the G+C mol% of each sequence set (the 16S rRNA sequence, the 10 genes and the concatenated sequences) in this study, compared to the genome-wide G+C mol%, ranked in increasing order. The G+C mol% of the *pgm* gene is closest to that of the total genome, while the 16S rRNA gene is the farthest. The notches are indicative of strong evidence that the medians differ when the notches do not overlap [51]. The 16S rRNA gene does not overlap with any other gene. In fact, a two-tailed *t-*test with a *P* value less than 0.001 (2.2×10^−16^) revealed that the G+C mol% of the 16S rRNA sequence was statistically distinct from that of the total genome G+C mol%. This indicates that the 16S rRNA gene is not matching the pace of drift of the total genome with regards to G+C mol%. In contrast, all of the glycolysis genes, with the exception of *pfk* and *eno*, were not statistically different from the total genome G+C mol% (*P* value greater than 0.01), indicating that G+C mol% drift for glycolysis genes provide insights into the genome-wide G+C mol% drift. This further supports glycolytic sequences as intriguing candidates for both phylogenetic studies and representatives of genome-wide trends.

**Fig. 7.**
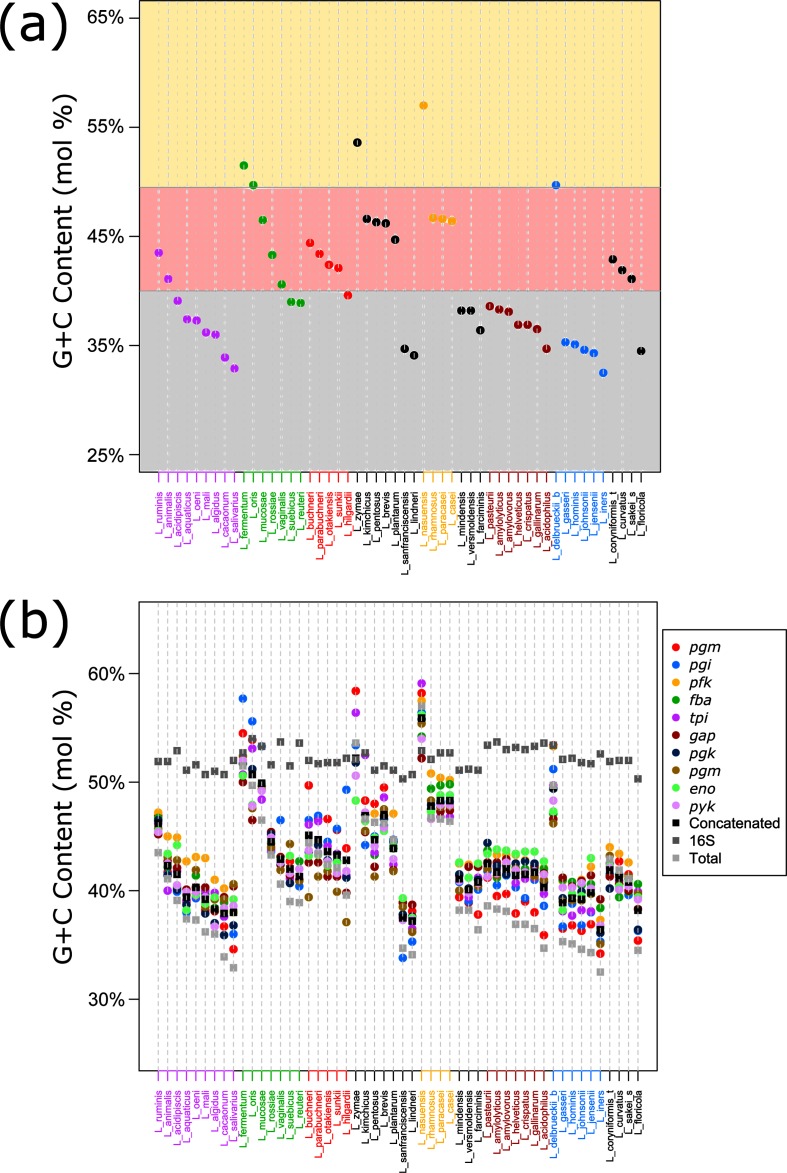
G+C mol% analysis of *Lactobacillus* genomes. (a) shows the total G+C mol% for each species. Species are coloured according to their phylogenetic group. (b) shows the G+C mol% of the glycolysis genes, the concatenated glycolysis genes, the 16S rRNA and total G+C mol% for each species. Species are named according to Table 1.

The genome sizes in this study ranged from 1.28 Mb (*Lactobacillus iners*) to 3.65 Mb (*Lactobacillus pentosus*), again reflecting the extensive genomic diversity within this genus. The total G+C mol% ranged from 32.50 % (*L. iners*) to 57.00 % (*Lactobacillus nasuensis*), which is intriguing given the general assumption that all lactobacilli are low G+C mol% organisms. Nevertheless, the mean G+C mol% was 40.70 %, consistent with *Lactobacillus* being generally perceived as low G+C mol% organisms. Splitting the species into high, medium and low categories, it becomes apparent that most species are trending towards the lower end of the spectrum, and away from the higher G+C mol% range (Fig. 7a). Some of the phylogenetic groups are closely clustered, such as the *L. acidophilus* group, *L. gasseri* group and the *L. rhamnosus* group, with the exception of *L. delbrueckii* subsp. *bulgaricus* (a dairy bacterium) and *L. nasuensis* (an aforementioned ex in G+C mol%). The *L. animalis* group and *L. buchneri* group are similarly clustered, albeit more loosely. These observations hold true when comparing the G+C mol% of all the individual genes in their respective genomes, perhaps reflecting a consistent and genome-wide pace of drift, rather than variable speeds of drift for each gene (Fig. 7b). Again, the 16S rRNA sequence has a much higher G+C mol% than most of the other studied genes, with the outlier *L. nasuensis* deviating from the consensus. The G+C mol% of the glycolysis genes within clusters are often times very close, as exemplified by the *L. acidophilus* group.

## Discussion

The genomic and functional attributes of *Lactobacillus* render it a pervasive genus, both in research and in industry. The benefits and uses of this diverse set of species are well-established and exhaustive, and yet, the list continues to grow. Many *Lactobacillus* strains are now considered to be health-promoting in the form of probiotics and are often found to be a part of a healthy microbiome [26]. They are also being engineered to promote healthy host–microbe interactions and deliver bioactive compounds such as vaccines [52]. As microbiome studies expand, we anticipate that the interest in *Lactobacillus* is set to increase, especially given their occurrence in several human-associated microbiomes, encompassing intestinal, vaginal, oral and skin communities [21]. Many studies have been published discussing the role of *Lactobacillus* in the microbiota, including research into the microbiota changes through disease, enhancing the microbiome as a form of treatment, and how the microbiome reacts to drugs [53–55]. The continuously expanding list of uses and studies just illustrates how important it is to accurately identify *Lactobacillus* species. While all species of *Lactobacillus* share some classical features of LAB organisms, notably their ability to produce lactic acid, the similarities between species are relatively few. In fact, even basic characteristics such as niche and isolation source can vary radically. Proper identification is an increasing concern especially when it comes to disease modelling in the human microbiome, as well as the formulation, tracking and efficacy of probiotic strains. Innovative techniques are continuously being developed and often use a combination of 16S rRNA with developing technologies, such as MALDI-TOF [56]. However, these tools are not broadly accessible and still rely partially on the sometimes unsatisfactory 16S rRNA. Here, we provide a practical alternative to the classical use of 16S rRNA sequencing.

In this paper, we applied the previously proposed methodology of using glycolysis sequences to perform phylogenetic studies [31] in the genus *Lactobacillus*. We demonstrated that this method is a practical and robust approach for *Lactobacillus*. Compared to the traditional 16S rRNA method, this approach was able to consistently identify phylogenetic groupings, with notably high-resolution between closely related species. While the 16S rRNA-based tree was able to identify the six phylogenetic groups, the concatenated tree was able to add more discrimination both between and within groups, evidenced by the higher bootstrap values in the glycolysis-based tree. Our grouping is consistent with a previous study using glycolysis sequences for phylogenetic analysis of *Lactobacillus* species [32]. Further analyses based on genomic content revealed clues as to why the glycolysis-based tree was better able to assign species.

First, looking at the organization of the genes in the genomes revealed two conserved operons in *Lactobacillus*, the *gap* operon and the *pfk* operon, with the remaining enzymes showing clear start and stop boundaries. This shared synteny emphasizes the importance of glycolysis gene conservation. Next, we looked at expression level. The glycolysis genes were consistently among the most highly expressed genes in the cell, with the *gap* gene always in the top three most abundant transcripts. These high expression levels indicate a great use and energy expenditure and, thus, arguably reflect the biological importance of this gene to the cell. Because of this importance, the glycolysis genes are much less likely to be subjected to loss. The operon structures and expression levels of the glycolysis genes are significant because a main criterion for selecting the 16S rRNA as a phylogenetic marker was its high conservation among species [57]. Next, we looked at how the glycolysis genes reflected genomic drift in terms of G+C mol%. First, it would appear that the genus is reaching a stabilizing point in its G+C mol% drift, though some species with high G+C mol% still have margin for extending the trend (*L. nasuensis, Lactobacillus zymae,* and *L. fermentum*). Next, we saw that the glycolysis gene G+C mol% was extremely close to that of the genome-wide G+C mol%, while the 16S rRNA was startlingly higher (*P*<0.001), underscoring the fact that the 16S rRNA is by all accounts much different than that of the total genome, whereas the majority of the glycolysis genes are significantly similar to the total genome G+C mol% (Fig. 6). This provides a possible explanation for the reason why the 16S rRNA analyses have been limited at a high-resolution level in *Lactobacillus* and why the glycolysis-based tree was able to reach a higher-resolution level. In fact, it has long been noted that 16S rRNA is unable to discriminate between species of lactobacilli due to its high similarity amongst them [58]. The individual glycolysis genes are much more similar to the genome as a whole (Fig. 6). Additionally, individual glycolysis genes are also able to accurately assign species to groups with a high resolution (Figs S4–S13). The *gap* gene is of particular note, due to its presence in an operon, consistently high expression, G+C mol% and ability to accurately define species groups. Overall, the glycolysis-based approach was able to provide a highe-resolution phylogeny for *Lactobacillus,* due in part to its conservation, expression and reflection of genomic drift.

## Supplementary Data

881 Supplementary File 1

## References

[1] Dandekar T, Snel B, Huynen M, Bork P (1998). Conservation of gene order: a fingerprint of proteins that physically interact. Trends Biochem Sci.

[2] Karlin S, Campbell AM, Mrázek J (1998). Comparative DNA analysis across diverse genomes. Annu Rev Genet.

[3] Karlin S, Mrázek J, Campbell A, Kaiser D (2001). Characterizations of highly expressed genes of four fast-growing bacteria. J Bacteriol.

[4] Boekhorst J, Siezen RJ, Zwahlen MC, Vilanova D, Pridmore RD (2004). The complete genomes of *Lactobacillus plantarum* and *Lactobacillus johnsonii* reveal extensive differences in chromosome organization and gene content. Microbiology.

[5] Hoffmann M, Zhao S, Pettengill J, Luo Y, Monday SR (2014). Comparative genomic analysis and virulence differences in closely related *Salmonella enterica* serotype eidelberg isolates from humans, retail meats, and animals. Genome Biol Evol.

[6] Losada PM, Tümmler B (2016). SNP synteny analysis of *Staphylococcus aureus* and *Pseudomonas aeruginosa* population genomics. FEMS Microbiol Lett.

[7] Makarova K, Slesarev A, Wolf Y, Sorokin A, Mirkin B (2006). Comparative genomics of the lactic acid bacteria. Proc Natl Acad Sci USA.

[8] Claesson MJ, van Sinderen D, O'Toole PW (2008). *Lactobacillus* phylogenomics-towards a reclassification of the genus. Int J Syst Evol Microbiol.

[9] Felis GE, Dellaglio F, Mizzi L, Torriani S (2001). Comparative sequence analysis of a *recA* gene fragment brings new evidence for a change in the taxonomy of the *Lactobacillus casei* group. Int J Syst Evol Microbiol.

[10] Milani C, Turroni F, Duranti S, Lugli GA, Mancabelli L (2016). Genomics of the genus *Bifidobacterium* reveals species-specific adaptation to the glycan-rich gut environment. Appl Environ Microbiol.

[11] Milani C, Lugli GA, Turroni F, Mancabelli L, Duranti S (2014). Evaluation of bifidobacterial community composition in the human gut by means of a targeted amplicon sequencing (ITS) protocol. FEMS Microbiol Ecol.

[12] Baker GC, Smith JJ, Cowan DA (2003). Review and re-analysis of domain-specific 16S primers. J Microbiol Methods.

[13] Clarridge JE (2004). Impact of 16S rRNA gene sequence analysis for identification of bacteria on clinical microbiology and infectious diseases. Clin Microbiol Rev.

[14] de La Cuesta-Zuluaga J, Escobar JS (2016). Considerations for optimizing microbiome analysis using a marker gene. Front Nutr.

[15] Salvetti E, Torriani S, Felis GE (2012). The genus *Lactobacillus*: a taxonomic update. Probiotics Antimicrob Proteins.

[16] Sun Z, Harris HM, McCann A, Guo C, Argimón S (2015). Expanding the biotechnology potential of lactobacilli through comparative genomics of 213 strains and associated genera. Nat Commun.

[17] Bernardeau M, Vernoux JP, Henri-Dubernet S, Guéguen M (2008). Safety assessment of dairy microorganisms: the *Lactobacillus* genus. Int J Food Microbiol.

[18] Hill C, Guarner F, Reid G, Gibson GR, Merenstein DJ (2014). Expert consensus document. The International Scientific Association for Probiotics and Prebiotics consensus statement on the scope and appropriate use of the term probiotic. Nat Rev Gastroenterol Hepatol.

[19] Saxelin M (2008). Probiotic formulations and applications, the current probiotics market, and changes in the marketplace: a European perspective. Clin Infect Dis.

[20] Cho I, Blaser MJ (2012). The human microbiome: at the interface of health and disease. Nat Rev Genet.

[21] Human Microbiome Project Consortium (2012). Structure, function and diversity of the healthy human microbiome. Nature.

[22] Conlon M, Bird A (2015). The impact of diet and lifestyle on gut microbiota and human health. Nutrients.

[23] Li X, Wang N, Yin B, Fang D, Jiang T (2016). Effects of *Lactobacillus plantarum* CCFM0236 on hyperglycaemia and insulin resistance in high-fat and streptozotocin-induced type 2 diabetic mice. J Appl Microbiol.

[24] Feng XB, Jiang J, Li M, Wang G, You JW (2016). Role of intestinal flora imbalance in pathogenesis of pouchitis. Asian Pac J Trop Med.

[25] Hooper LV, Midtvedt T, Gordon JI (2002). How host-microbial interactions shape the nutrient environment of the mammalian intestine. Annu Rev Nutr.

[26] Gerritsen J, Smidt H, Rijkers GT, de Vos WM (2011). Intestinal microbiota in human health and disease: the impact of probiotics. Genes Nutr.

[27] Shreiner AB, Kao JY, Young VB (2015). The gut microbiome in health and in disease. Curr Opin Gastroenterol.

[28] Okai S, Usui F, Yokota S, Hori-I Y, Hasegawa M (2016). High-affinity monoclonal IgA regulates gut microbiota and prevents colitis in mice. Nat Microbiol.

[29] O'Flaherty S, Klaenhammer TR (2016). Multivalent chromosomal expression of the *Clostridium botulinum* serotype a neurotoxin heavy-chain antigen and the *Bacillus anthracis* protective antigen in *Lactobacillus acidophilus*. Appl Environ Microbiol.

[30] Hug LA, Baker BJ, Anantharaman K, Brown CT, Probst AJ (2016). A new view of the tree of life. Nat Microbiol.

[31] Brandt K, Barrangou R (2016). Phylogenetic analysis of the *Bifidobacterium* genus using glycolysis enzyme sequences. Front Microbiol.

[32] Salvetti E, Fondi M, Fani R, Torriani S, Felis GE (2013). Evolution of lactic acid bacteria in the order *Lactobacillales* as depicted by analysis of glycolysis and pentose phosphate pathways. Syst Appl Microbiol.

[33] Fothergill-Gilmore LA (1986). The evolution of the glycolytic pathway. Trends Biochem Sci.

[34] Fothergill-Gilmore LA, Michels PA (1993). Evolution of glycolysis. Prog Biophys Mol Biol.

[35] Kearse M, Moir R, Wilson A, Stones-Havas S, Cheung M (2012). Geneious Basic: an integrated and extendable desktop software platform for the organization and analysis of sequence data. Bioinformatics.

[36] Altschul SF, Gish W, Miller W, Myers EW, Lipman DJ (1990). Basic local alignment search tool. J Mol Biol.

[37] Contreras-Moreira B, Vinuesa P (2013). GET_HOMOLOGUES, a versatile software package for scalable and robust microbial pangenome analysis. Appl Environ Microbiol.

[38] Gasteiger E, Gattiker A, Hoogland C, Ivanyi I, Appel RD (2003). ExPASy: the proteomics server for in-depth protein knowledge and analysis. Nucleic Acids Res.

[39] Johnson BR, Hymes J, Sanozky-Dawes R, Henriksen ED, Barrangou R (2016). Conserved S-layer-associated proteins revealed by exoproteomic survey of S-layer-forming lactobacilli. Appl Environ Microbiol.

[40] Katoh K, Misawa K, Kuma K, Miyata T (2002). MAFFT: a novel method for rapid multiple sequence alignment based on fast Fourier transform. Nucleic Acids Res.

[41] Edgar RC (2004). MUSCLE: multiple sequence alignment with high accuracy and high throughput. Nucleic Acids Res.

[42] Larkin MA, Blackshields G, Brown NP, Chenna R, McGettigan PA (2007). Clustal W and Clustal X version 2.0. Bioinformatics.

[43] Capella-Gutiérrez S, Silla-Martínez JM, Gabaldón T (2009). trimAl: a tool for automated alignment trimming in large-scale phylogenetic analyses. Bioinformatics.

[44] Stamatakis A (2014). RAxML version 8: a tool for phylogenetic analysis and post-analysis of large phylogenies. Bioinformatics.

[45] Core Team R (2015). R: A Language and Environment for Statistical Computing.

[46] Canchaya C, Claesson MJ, Fitzgerald GF, van Sinderen D, O'Toole PW (2006). Diversity of the genus *Lactobacillus* revealed by comparative genomics of five species. Microbiology.

[47] Heinl S, Wibberg D, Eikmeyer F, Szczepanowski R, Blom J (2012). Insights into the completely annotated genome of *Lactobacillus buchneri* CD034, a strain isolated from stable grass silage. J Biotechnol.

[48] Cárdenas N, Laiño JE, Delgado S, Jiménez E, Juárez del Valle M (2015). Relationships between the genome and some phenotypical properties of *Lactobacillus fermentum* CECT 5716, a probiotic strain isolated from human milk. Appl Microbiol Biotechnol.

[49] Arsköld E, Lohmeier-Vogel E, Cao R, Roos S, Rådström P (2008). Phosphoketolase pathway dominates in *Lactobacillus reuteri* ATCC 55730 containing dual pathways for glycolysis. J Bacteriol.

[50] Broadbent JR, Hughes JE, Welker DL, Tompkins TA, Steele JL (2013). Complete genome sequence for *Lactobacillus helveticus* CNRZ 32, an industrial cheese starter and cheese flavor adjunct. Genome Announc.

[51] Chambers JM (1983). Notched box plots. GraphicalMethods for Data Analysis.

[52] Seegers JF (2002). Lactobacilli as live vaccine delivery vectors: progress and prospects. Trends Biotechnol.

[53] Bhat M, Arendt BM, Bhat V, Renner EL, Humar A (2016). Implication of the intestinal microbiome in complications of cirrhosis. World J Hepatol.

[54] Bull-Otterson L, Feng W, Kirpich I, Wang Y, Qin X (2013). Metagenomic analyses of alcohol induced pathogenic alterations in the intestinal microbiome and the effect of *Lactobacillus rhamnosus* GG treatment. PLoS One.

[55] Shin CM, Kim N, Kim YS, Nam RH, Park JH (2016). Impact of Long-term proton pump inhibitor therapy on gut microbiota in F344 rats: pilot study. Gut Liver.

[56] Foschi C, Laghi L, Parolin C, Giordani B, Compri M (2017). Novel approaches for the taxonomic and metabolic characterization of lactobacilli: integration of 16S rRNA gene sequencing with MALDI-TOF MS and 1H-NMR. PLoS One.

[57] Eisen JA (1995). The RecA protein as a model molecule for molecular systematic studies of bacteria: comparison of trees of RecAs and 16S rRNAs from the same species. J Mol Evol.

[58] Fox GE, Wisotzkey JD, Jurtshuk P (1992). How close is close: 16S rRNA sequence identity may not be sufficient to guarantee species identity. Int J Syst Bacteriol.

