# Supplementary material for: Using glycolysis enzyme sequences to inform Lactobacillus phylogeny
Source: Microb Genom. 2018 Jun 22;4(6):e000187. doi: 10.1099/mgen.0.000187 (PMC6096939; doi:10.1099/mgen.0.000187)
Supplement: Supplementary File 1 [file mgen-4-187-s001.pdf]

Tree	E.C. Number	Sum
16S		146.902
Concatenated		101.7221
Phosphoglucomutase, (*pgm*)	5.4.2.2	111.9324
Glucose-6-phosphate isomerase, (*pgi*)	5.3.1.9	118.2525
6-phosphofructokinase, (*pfk*)	2.7.1.11	69.89766
Fructose-bisphosphate aldolase, (*fba*)	4.1.2.13	86.18618
Triosephosphate isomerase, (*tpi*)	5.3.1.1	152.2155
Glyceraldehyde 3-phosphate dehydrogenase, (*gap*)	1.2.1.12	104.6662
Phosphoglycerate kinase, (*pgk*)	2.7.2.3	109.189
Phosphoglycerate mutase, (*gpm*)	5.4.2.11	243.8897
Enolase, (*eno*)	4.2.1.11	113.4122
Pyruvate kinase, (*pyk*)	2.7.1.40	106.5558

Table S1. Sum of Branch Lengths. The sum of branch lengths for each tree can be found in this table. Included are abbreviations for each glycolysis gene and their E.C. number.

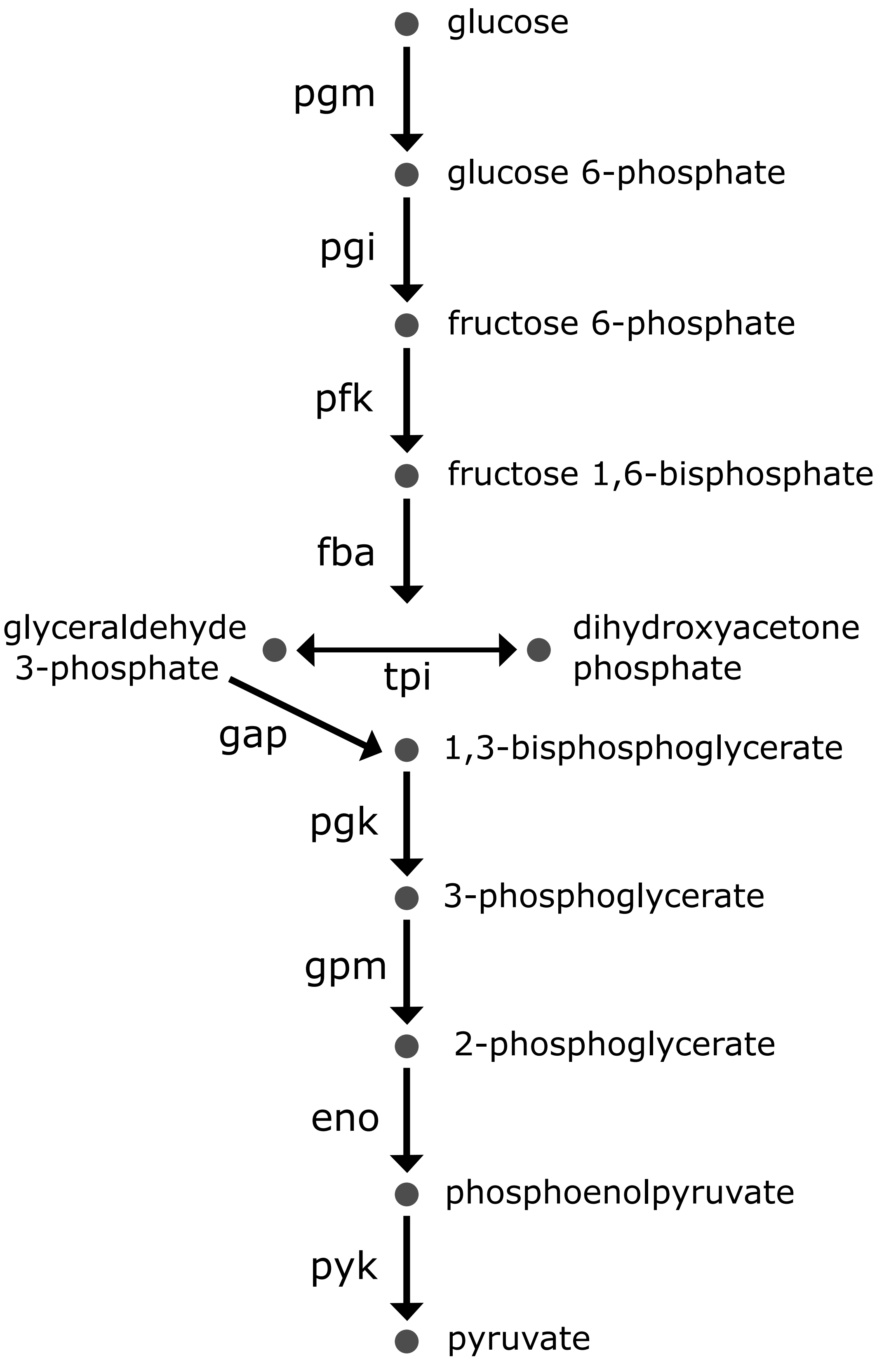

Fig. S1: Glycolysis Pathway in *Lactobacillus*. Depicted is the glycolysis pathway. Products are to the right of the dots and the enzymes used in this study are to the left of the arrows. Enzyme abbreviations can be found in Table S1.

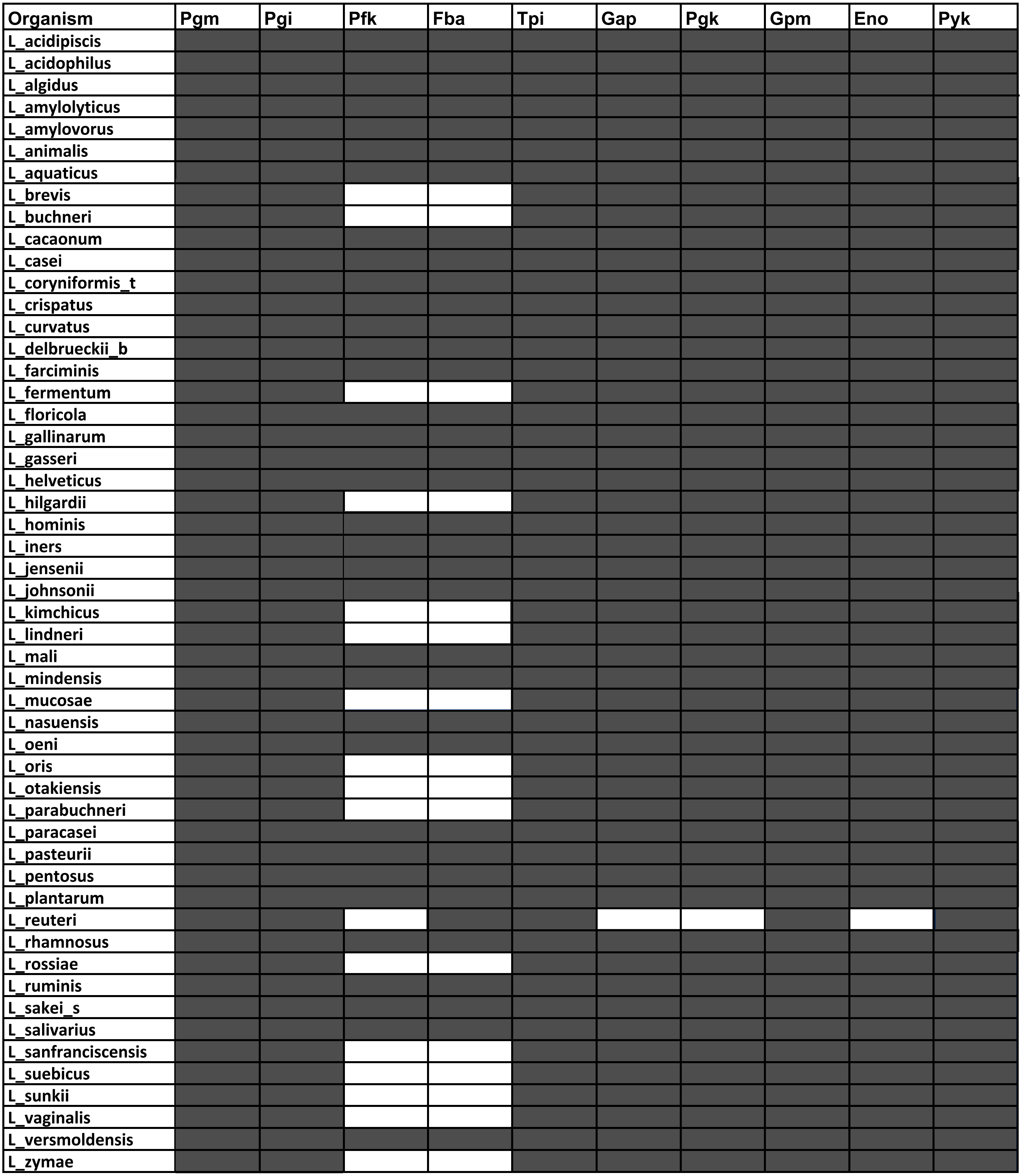

Fig. S2. Glycolysis Presence in *Lactobacillus.* Shows the presence of each glycolysis gene in each species of this study. Absence is reflected by a white box. Species follow the naming convention from Table 1. Gene abbreviations can be found in Supplemental Table 1.

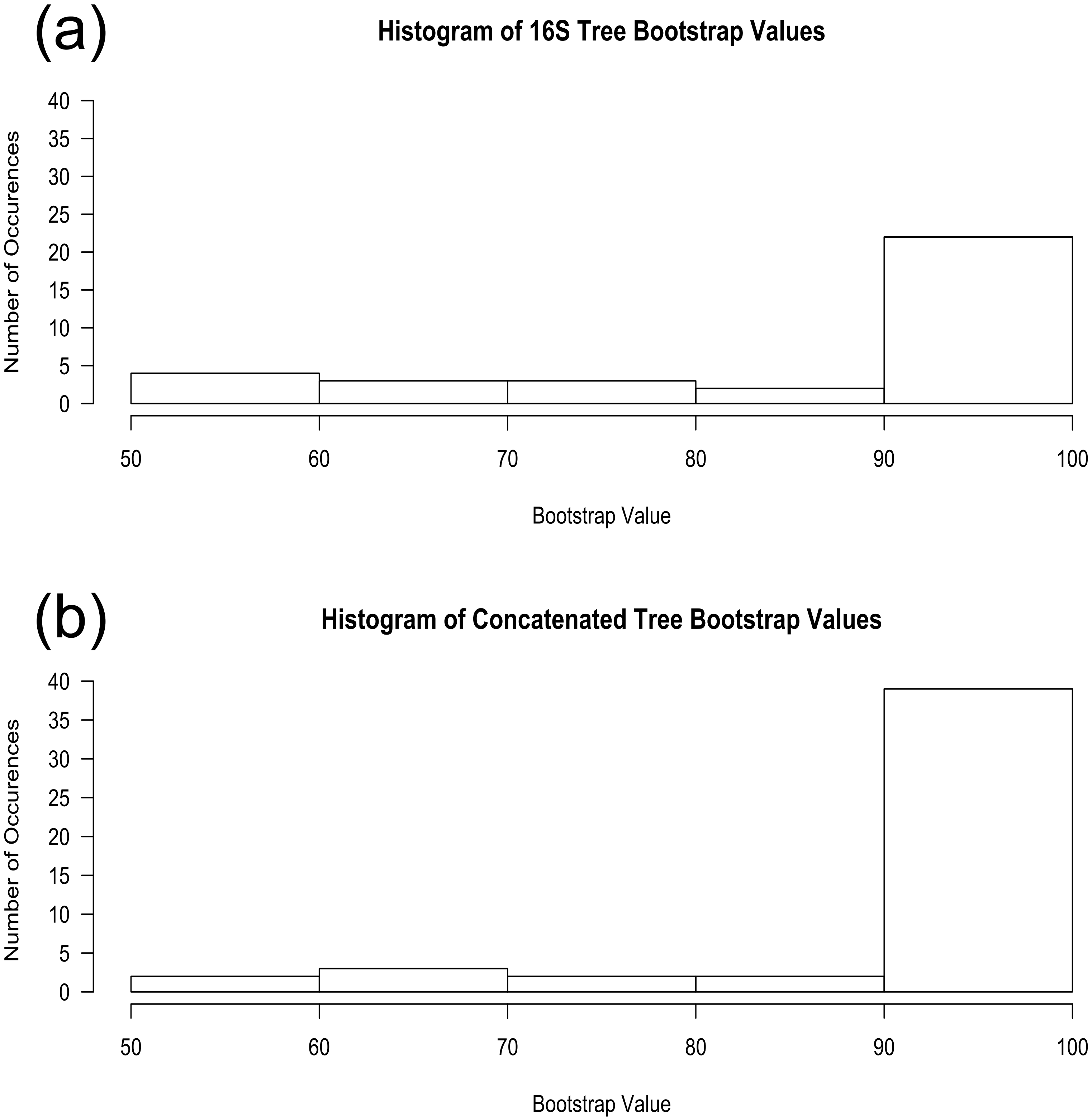

Fig. S3: Histogram of Bootstrap Values. (a) shows the bootstrap values for the 16S based trees. (b) shows the bootstrap values for the Glycolysis-based Concatenated Tree.

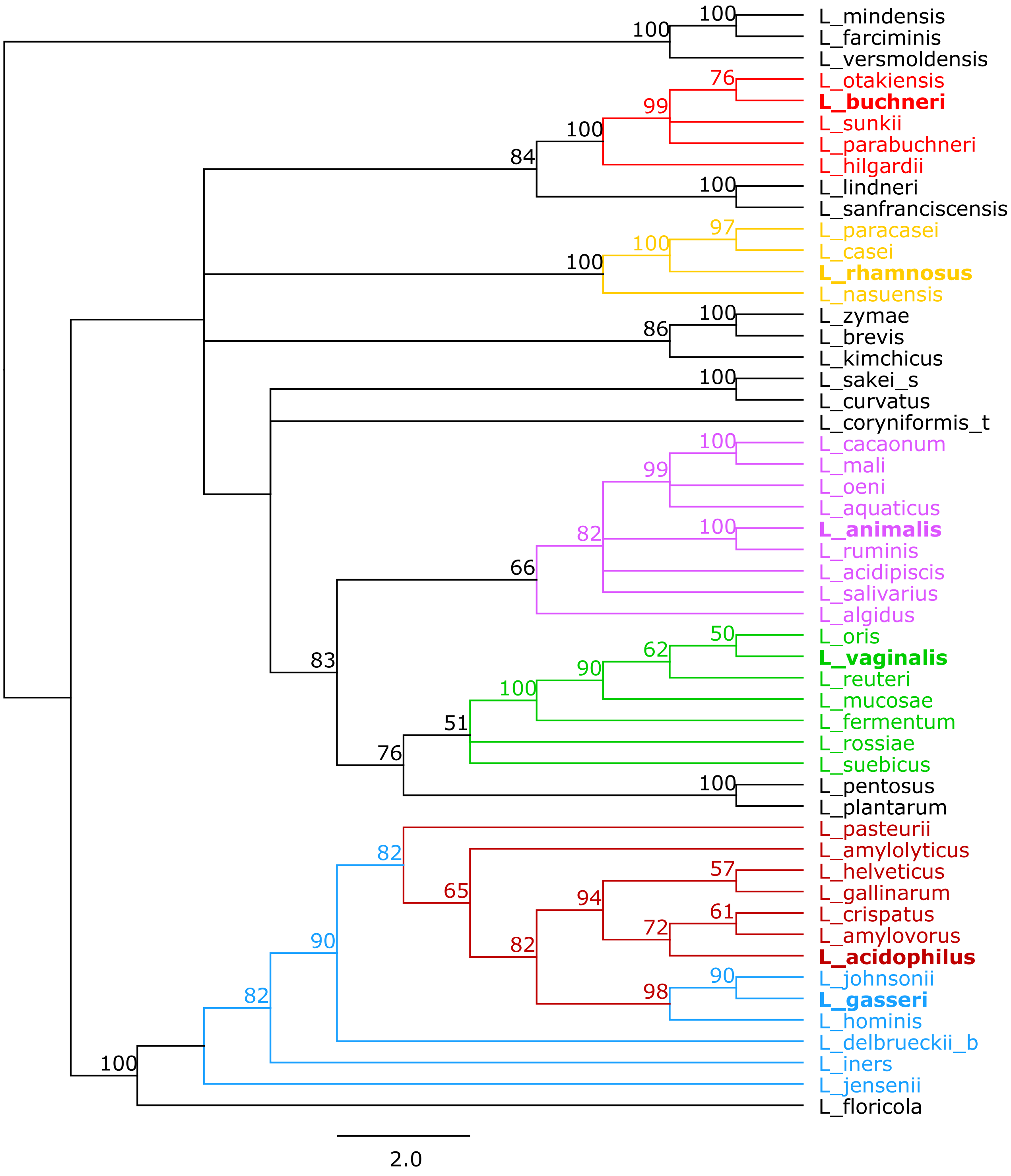

Fig. S4. Pgm Tree. Tree based off of the alignment of amino acid sequences of Pgm using RaxML. Bootstrap values are recorded on the nodes. Groups are colored as follows: the *L. animalis* group in purple, the *L. vaginalis* group in green, *L. buchneri* group in red, the *L. rhamnosus* group in yellow, the *L. acidophilus* group in maroon, the *L. gasseri* group in blue. The representative species in each group is in bold. Species name follows the naming convention in Table 1.

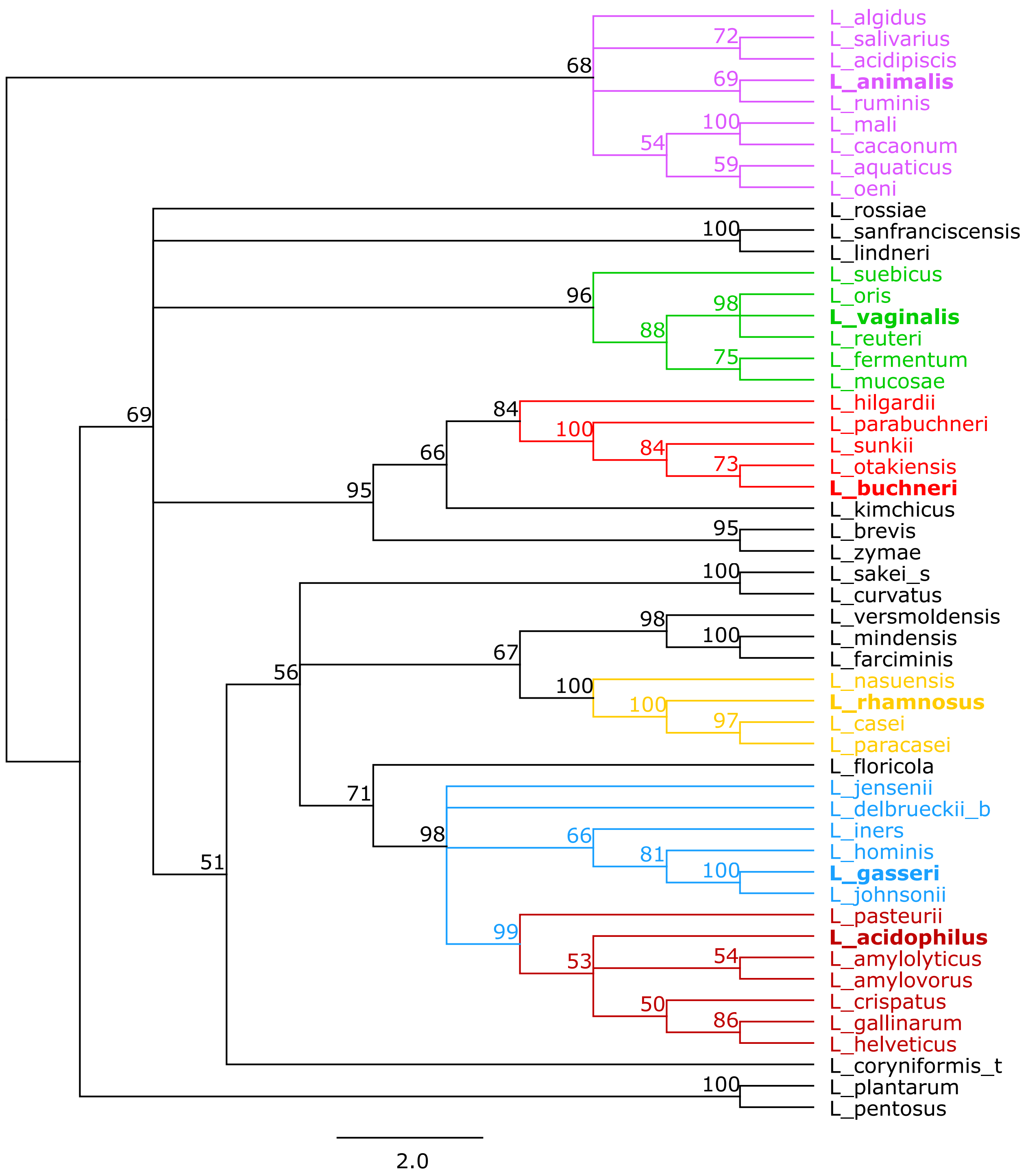

Fig. S5. Pgi Tree. Tree based off of the alignment of amino acid sequences of Pgi using RaxML. Bootstrap values are recorded on the nodes. Groups are colored as follows: the *L. animalis* group in purple, the *L. vaginalis* group in green, *L. buchneri* group in red, the *L. rhamnosus* group in yellow, the *L. acidophilus* group in maroon, the *L. gasseri* group in blue. The representative species in each group is in bold. Species name follows the naming convention in Table 1.

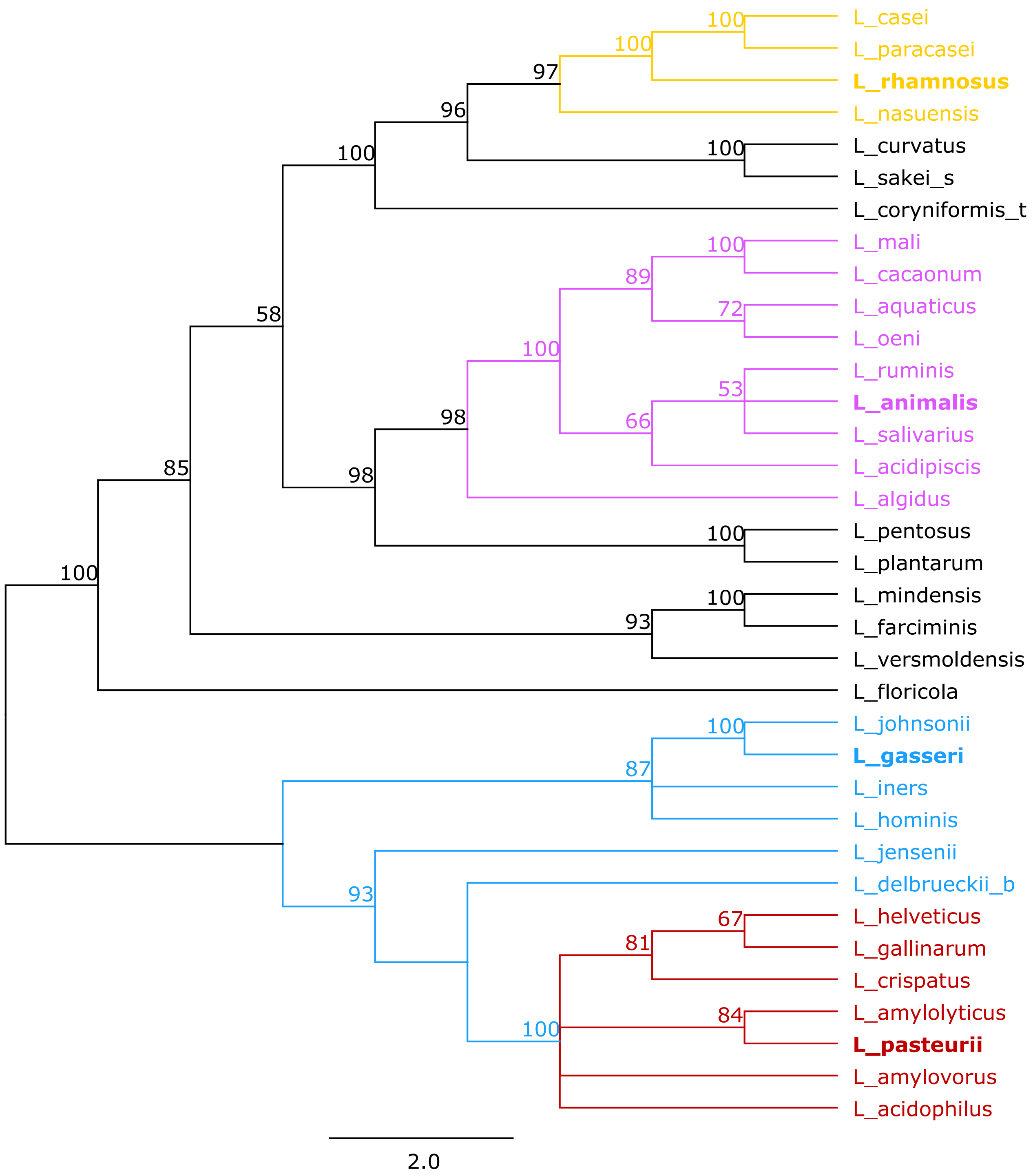

Fig. S6. Pfk Tree. Tree based off of the alignment of amino acid sequences of Pfk using RaxML. Bootstrap values are recorded on the nodes. Groups are colored as follows: the *L. animalis* group in purple, the *L. rhamnosus* group in yellow, the *L. acidophilus* group in maroon, the *L. gasseri* group in blue. The representative species in each group is in bold. Species name follows the naming convention in Table 1.

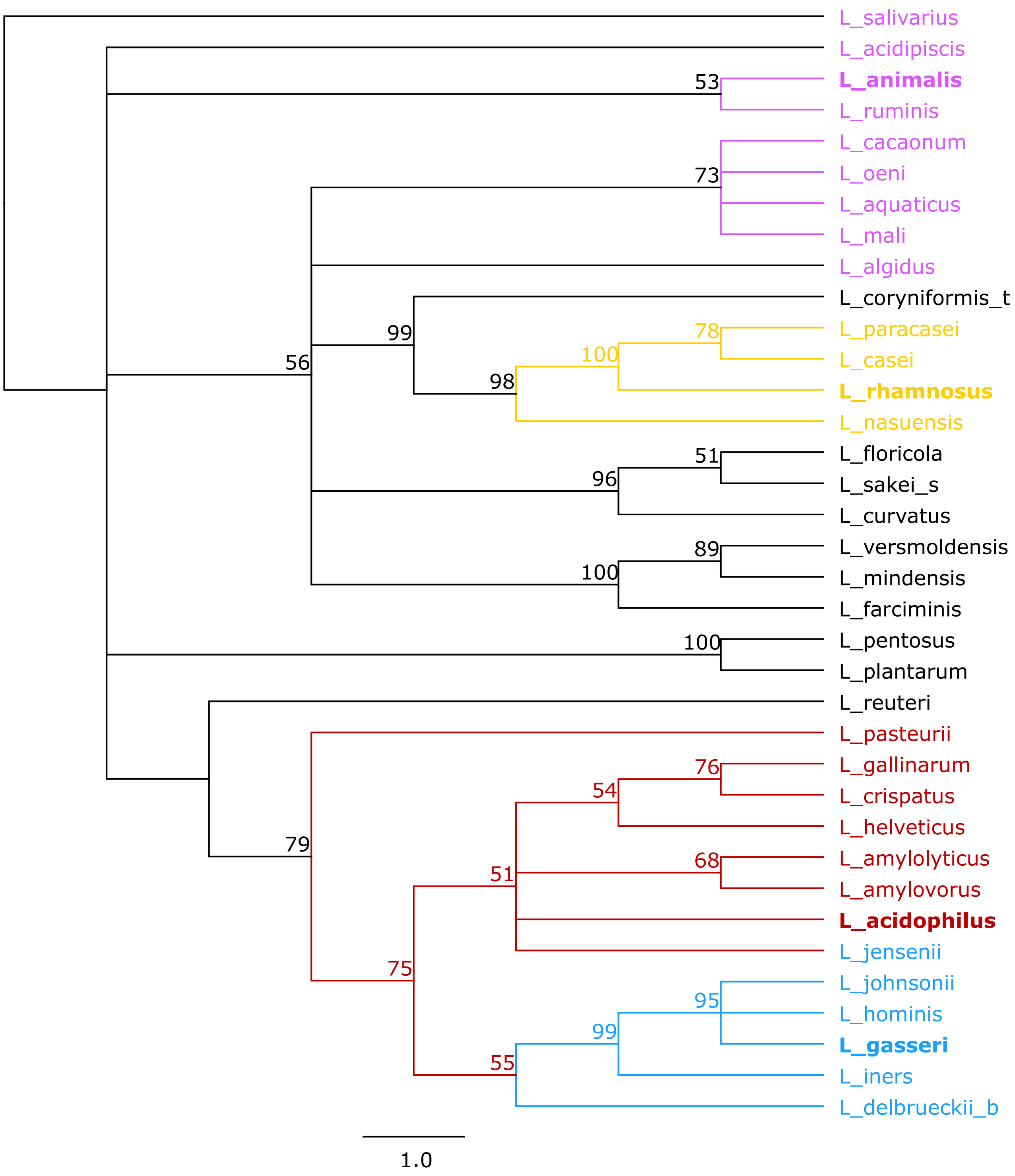

Fig. S7. Fba Tree. Tree based off of the alignment of amino acid sequences of Fba using RaxML. Bootstrap values are recorded on the nodes. Groups are colored as follows: the *L. animalis* group in purple, the *L. rhamnosus* group in yellow, the *L. acidophilus* group in maroon, the *L. gasseri* group in blue. The representative species in each group is in bold. Species name follows the naming convention in Table 1.

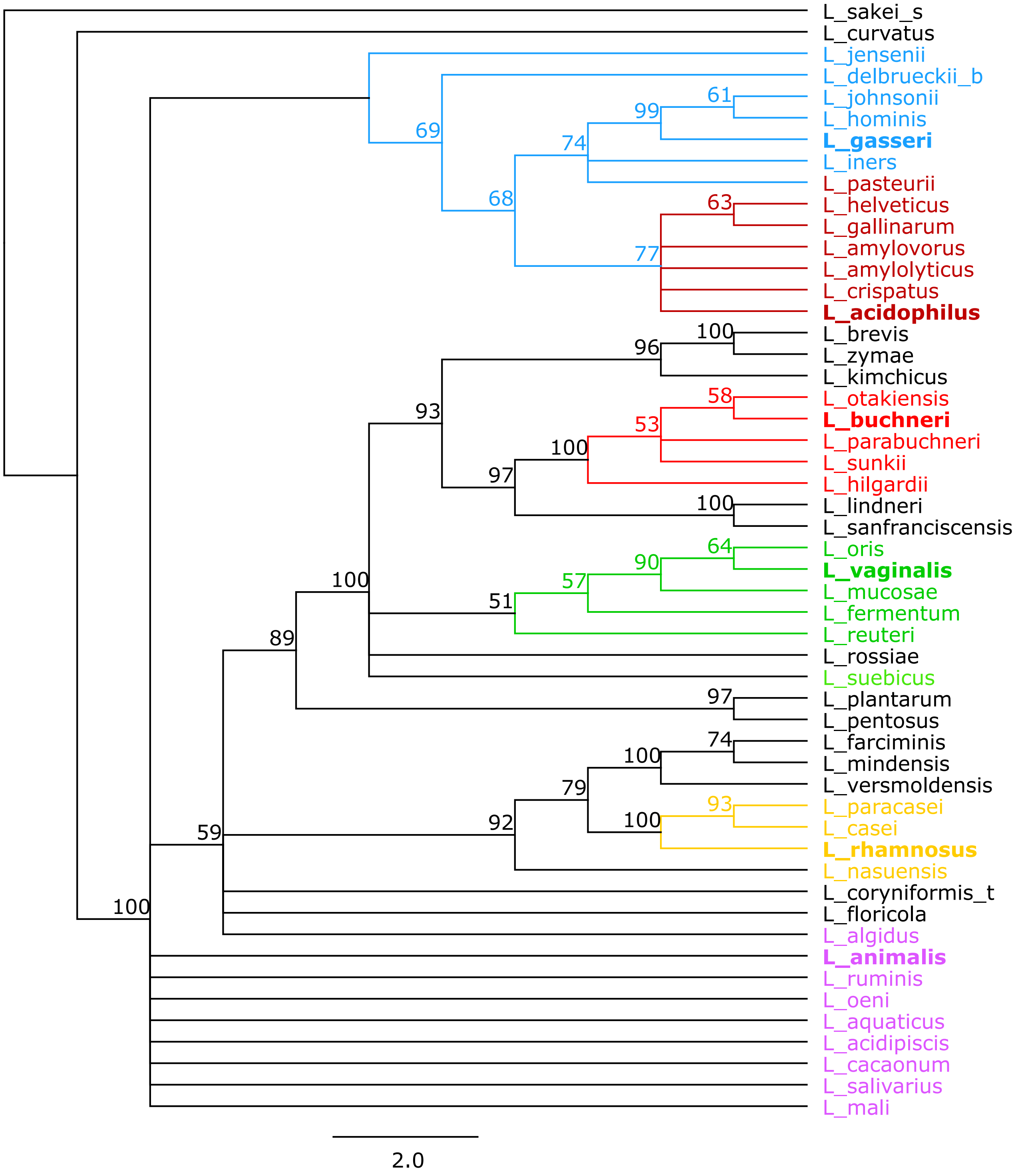

Fig. S8. Tpi Tree. Tree based off of the alignment of amino acid sequences of Tpi using RaxML. Bootstrap values are recorded on the nodes. Groups are colored as follows: the *L. animalis* group in purple, the *L. vaginalis* group in green, *L. buchneri* group in red, the *L. rhamnosus* group in yellow, the *L. acidophilus* group in maroon, the *L. gasseri* group in blue. The representative species in each group is in bold. Species name follows the naming convention in Table 1.

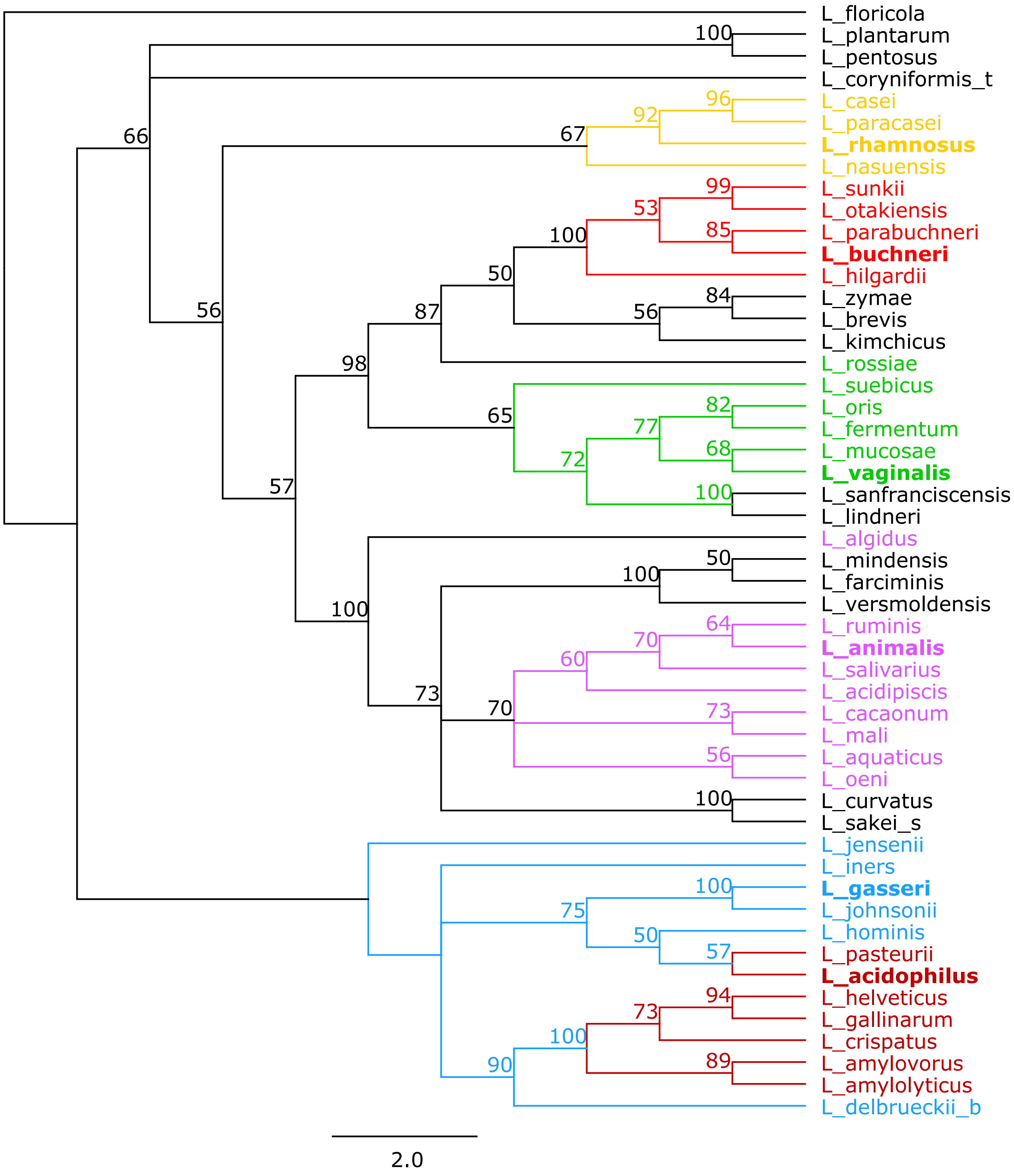

Fig. S9. Gap Tree. Tree based off of the alignment of amino acid sequences of Gap using RaxML. Bootstrap values are recorded on the nodes. Groups are colored as follows: the *L. animalis* group in purple, the *L. vaginalis* group in green, *L. buchneri* group in red, the *L. rhamnosus* group in yellow, the *L. acidophilus* group in maroon, the *L. gasseri* group in blue. The representative species in each group is in bold. Species name follows the naming convention in Table 1.

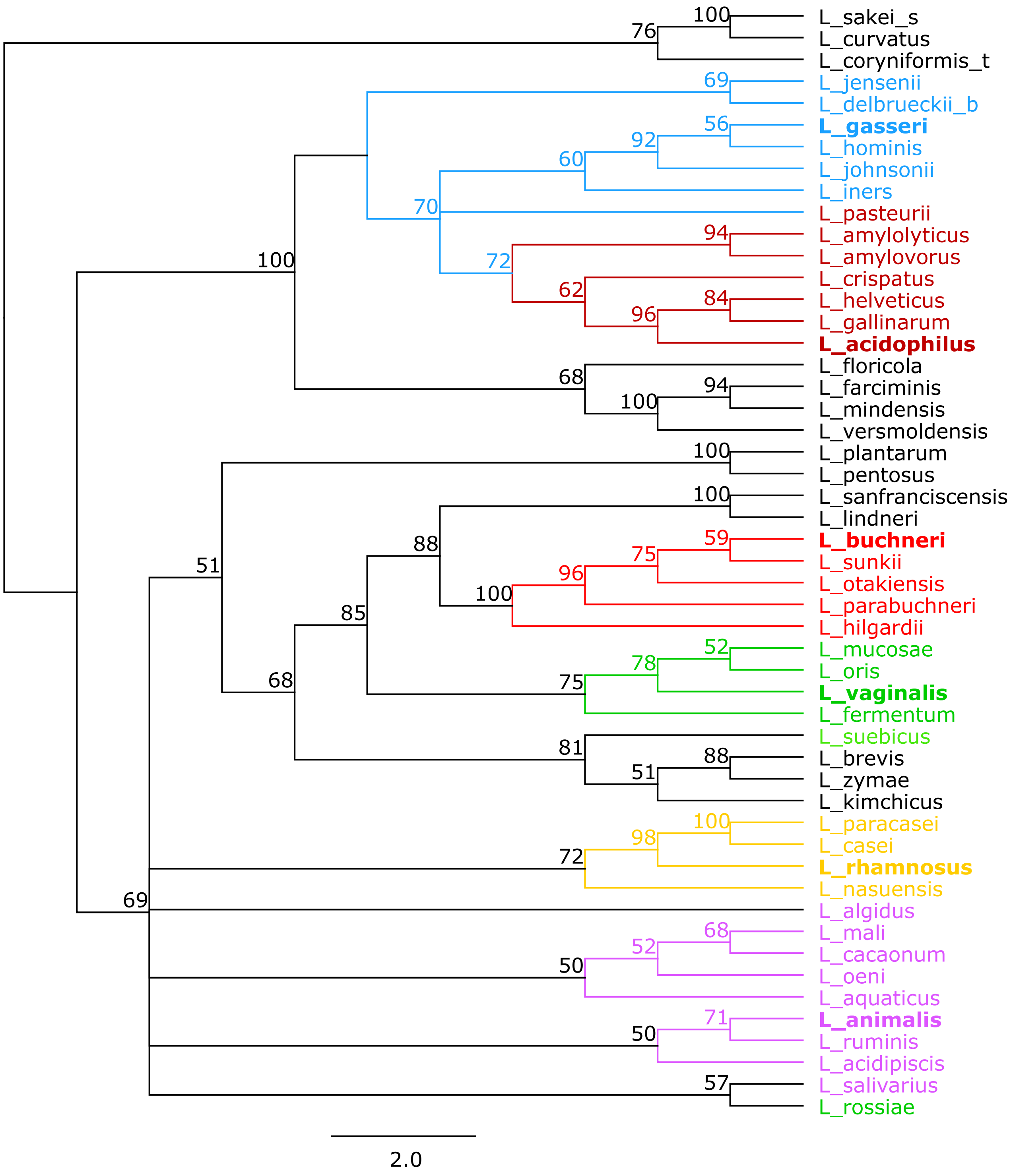

Fig. S10. Pgk Tree. Tree based off of the alignment of amino acid sequences of Pgk using RaxML. Bootstrap values are recorded on the nodes. Groups are colored as follows: the *L. animalis* group in purple, the *L. vaginalis* group in green, *L. buchneri* group in red, the *L. rhamnosus* group in yellow, the *L. acidophilus* group in maroon, the *L. gasseri* group in blue. The representative species in each group is in bold. Species name follows the naming convention in Table 1.

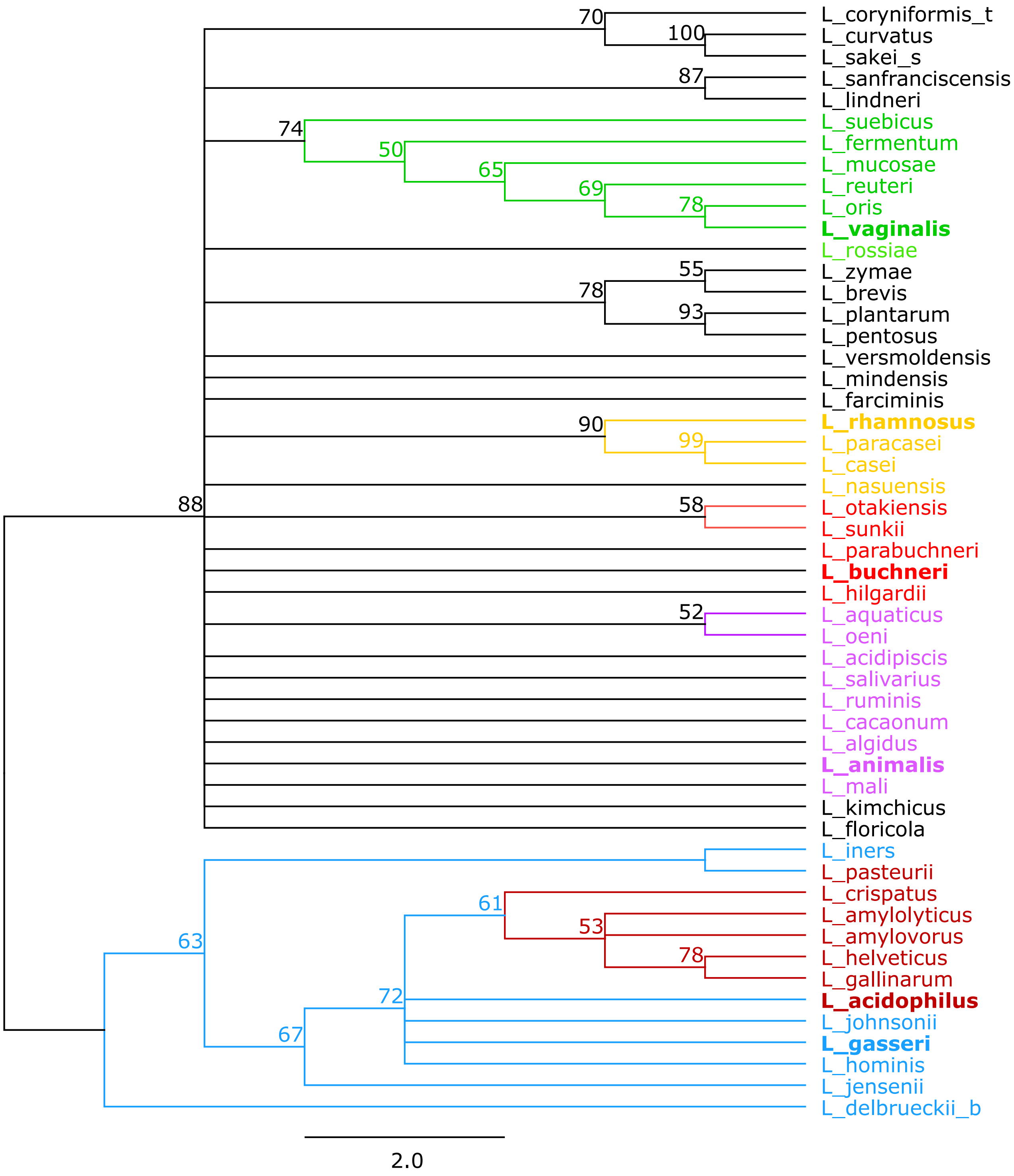

Fig. S11. Gpm Tree. Tree based off of the alignment of amino acid sequences of Gpm using RaxML. Bootstrap values are recorded on the nodes. Groups are colored as follows: the *L. animalis* group in purple, the *L. vaginalis* group in green, *L. buchneri* group in red, the *L. rhamnosus* group in yellow, the *L. acidophilus* group in maroon, the *L. gasseri* group in blue. The representative species in each group is in bold. Species name follows the naming convention in Table 1.

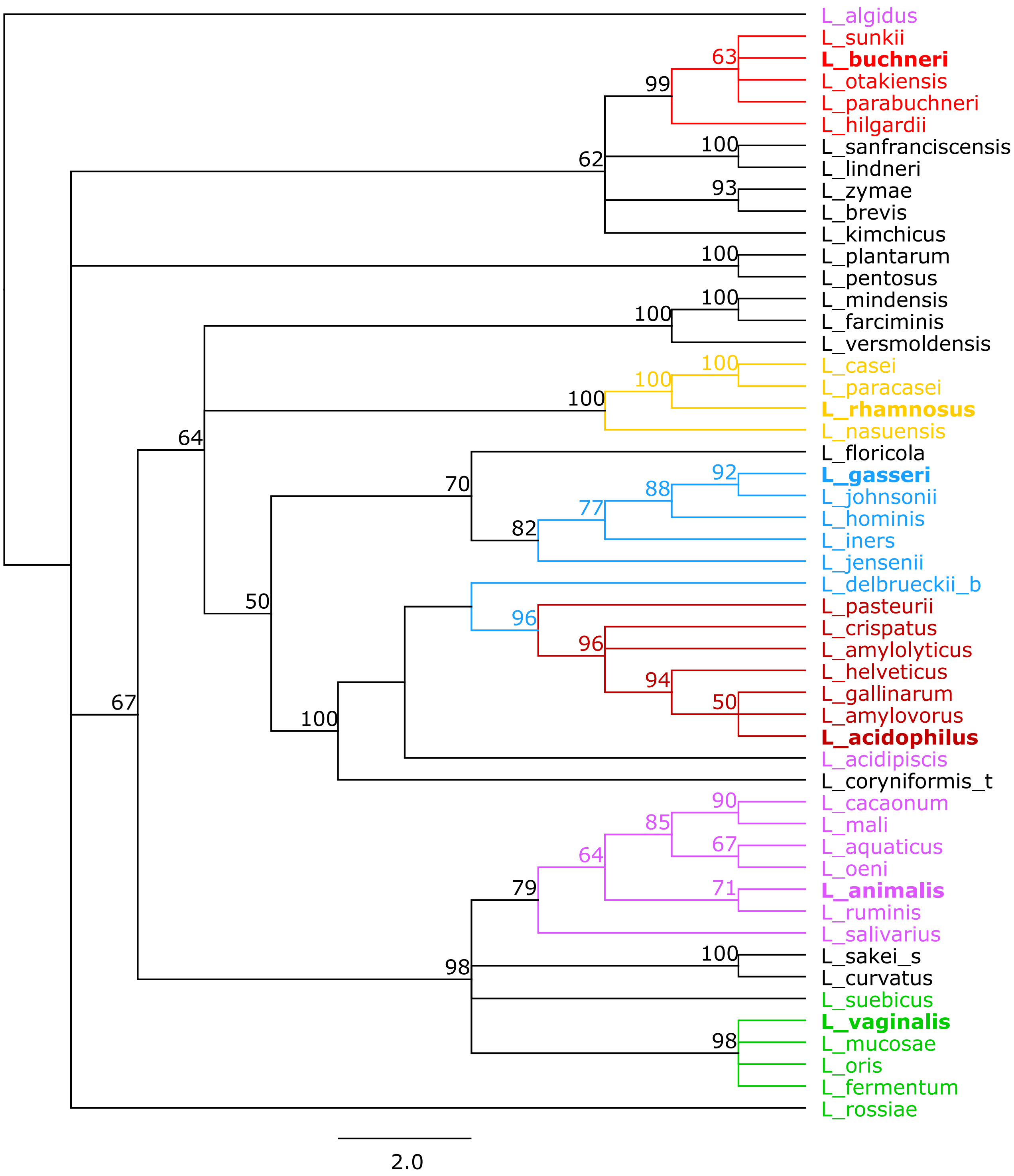

Fig. S12. Eno Tree. Tree based off of the alignment of amino acid sequences of Eno using RaxML. Bootstrap values are recorded on the nodes. Groups are colored as follows: the *L. animalis* group in purple, the *L. vaginalis* group in green, *L. buchneri* group in red, the *L. rhamnosus* group in yellow, the *L. acidophilus* group in maroon, the *L. gasseri* group in blue. The representative species in each group is in bold. Species name follows the naming convention in Table 1.

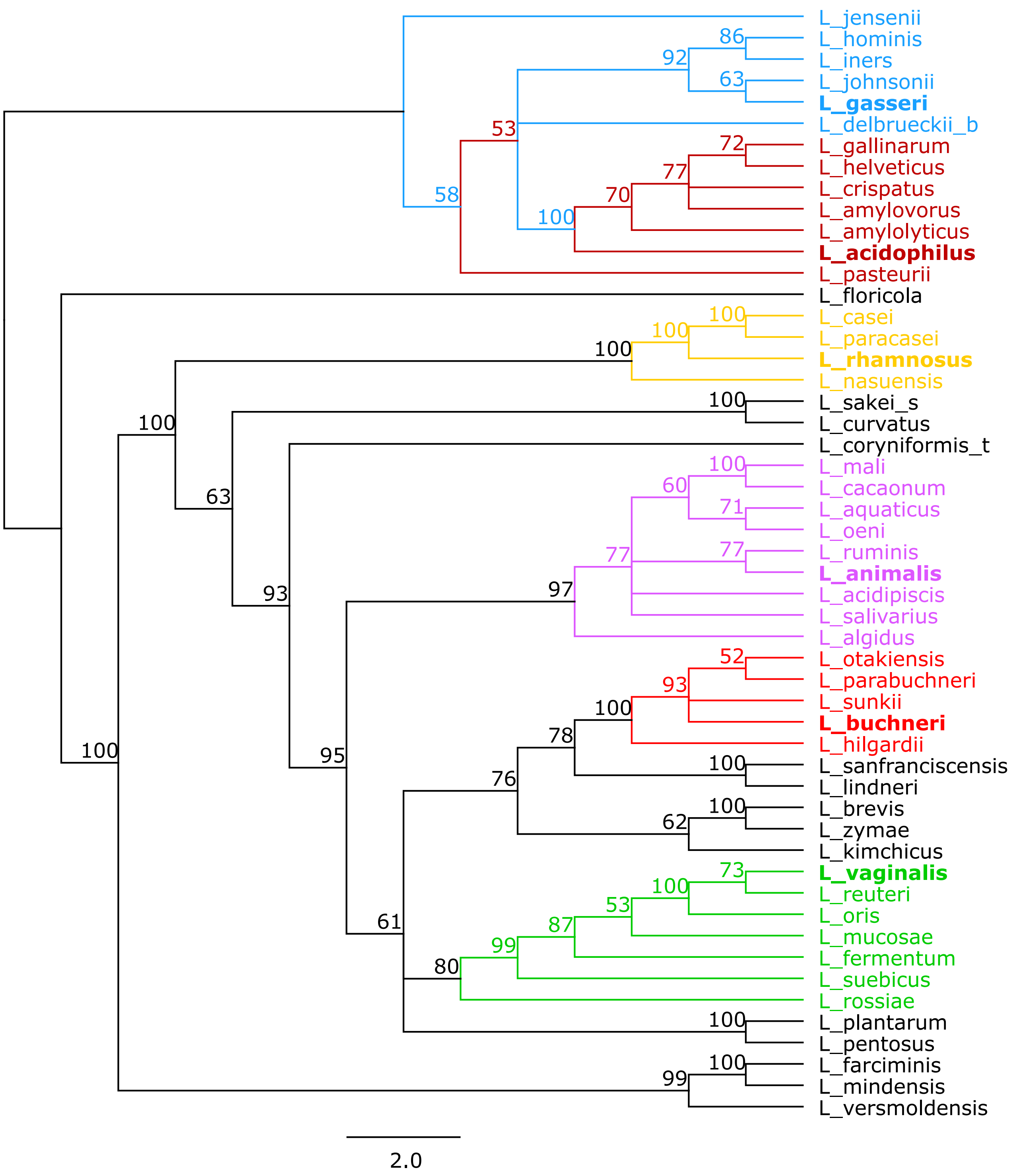

Fig. S13. Pyk Tree. Tree based off of the alignment of amino acid sequences of Pyk using RaxML. Bootstrap values are recorded on the nodes. Groups are colored as follows: the *L. animalis* group in purple, the *L. vaginalis* group in green, *L. buchneri* group in red, the *L. rhamnosus* group in yellow, the *L. acidophilus* group in maroon, the *L. gasseri* group in blue. The representative species in each group is in bold. Species name follows the naming convention in Table 1.
